# Autoregressive Modeling and Prediction of the Activity of Antihypertensive Peptides

**DOI:** 10.3389/fgene.2021.801728

**Published:** 2022-01-11

**Authors:** Xufen Xie, Chuanchuan Zhu, Di Wu, Ming Du

**Affiliations:** ^1^ School of Information Science and Engineering, Dalian Polytechnic University, Dalian, China; ^2^ School of Food Science and Technology, Dalian Polytechnic University, Dalian, China; ^3^ National Engineering Technology Research Center of Seafood, Dalian Polytechnic University, Dalian, China

**Keywords:** antihypertensive peptides, NARXNN, fractal characteristics, EC_50_ prediction, machine learning

## Abstract

Naturally derived bioactive peptides with antihypertensive activities serve as promising alternatives to pharmaceutical drugs. There are few relevant reports on the mapping relationship between the EC_50_ value of antihypertensive peptide activity (AHTPA-EC_50_) and its corresponding amino acid sequence (AAS) at present. In this paper, we have constructed two group series based on sorting natural logarithm of AHTPA-EC_50_ or sorting its corresponding AAS encoding number. One group possesses two series, and we find that there must be a random number series in any group series. The random number series manifests fractal characteristics, and the constructed series of sorting natural logarithm of AHTPA-EC_50_ shows good autocorrelation characteristics. Therefore, two non-linear autoregressive models with exogenous input (NARXs) were established to describe the two series. A prediction method is further designed for AHTPA-EC_50_ prediction based on the proposed model. Two dynamic neural networks for NARXs (NARXNNs) are designed to verify the two series characteristics. Dipeptides and tripeptides are used to verify the proposed prediction method. The results show that the mean square error (MSE) of prediction is about 0.5589 for AHTPA-EC_50_ prediction when the classification of AAS is correct. The proposed method provides a solution for AHTPA-EC_50_ prediction.

## 1 Introduction

Hypertension is a clinical syndrome characterized by increased systemic arterial blood pressure, which can be accompanied by functional or organic damage of the heart, brain, kidney, and other organs. The renin–angiotensin system (RAS) controls blood pressure by regulating the volume of blood in blood vessels. The angiotensin-converting enzyme (ACE) is the core component of the RAS. The ACE can convert inactive angiotensin I into angiotensin II with vasoconstriction, which indirectly increases blood pressure (Zhang et al., 2000). Therefore, ACE inhibitors are widely used as drugs for the treatment of cardiovascular diseases (Stone, 2018). Antihypertensive active peptide is an effective ACE inhibitor (Tu et al, 2018a; Tu et al, 2018b; Wu et al, 2019), which has attracted great attention in the treatment and prevention of hypertension. The EC_50_ value (sample concentration when the ACE inhibition rate is 50%) describes the activity of antihypertensive peptide, which is the most important index to select antihypertensive active peptide. Some research studies focus on feature representation (Tong, et al, 2008; Manavalan et al, 2019), and some research studies focus on identification (Majumder, and Wu, 2010). Machine learning (ML) approaches are becoming more and more popular in bioinformatics (Baldi et al., 2001; Libbrecht and Noble, 2015; Zou and Qiliu, 2019; Yang et al., 2020; Zhang et al., 2021). Some research studies are associated with classification, and some are associated with regression. In 2015, Kumar et al. developed four different model types for predicting AHTPs with varied lengths using ML approaches (Kumar et al., 2015a; Kumar et al., 2015b). Another paper on AHTP prediction used random forest (RF) approaches (Win et al., 2018). However, there is great uncertainty in the relationship between the AAS of antihypertensive peptides and its corresponding AHTPA-EC_50_. So far, the mapping relationship between AHTPA-EC_50_ and its corresponding AAS has not been reported. The existing published data show that AHTPA-EC_50_ has multi-scale characteristics. It is difficult to establish a deterministic model between the AAS and AHTPA-EC_50_ directly.

Fractal phenomena generally exist in nature. Fractal data have the characteristics of instability, self-similarity, and multi-scale (Ruderman, 1996; Ghosh and Somvanshi, 2008; Al-Hamdan, et al, 2010; Al-Hamdan et al, 2012). The spectrum of fractal data is consistent (Pentland, 1984; Nill and Bouzas, 1992; Wornell and Oppenheim, 1992). These characteristics can be used to describe physical phenomena with statistical fractal. Fractional Brownian motion (FBM) (Chow, 2011; Kim and Kim, 2004; Fouché and Mukeru, 2013) is more universal than ordinary Brownian motion, and it can better describe the fractal phenomena in nature. FBM can be modeled and described by the time series of dynamic system, and time-series analysis is an important method of system identification and analysis. Yule first proposed the autoregressive (AR) model to predict the law of market change in 1927. In the 1960s, time-series analysis made a great progress in spectral analysis and estimation. The research of linear time-series model has been greatly developed from the AR model to autoregressive moving average (ARMA) modeling theory. Engle and Granger developed estimation procedures, tests, and empirical examples for the relationship between co-integration and error correction models (Engle and Granger 1987), and Hannan and Deistler proposed the multivariable VARMA model and VARMAX model (Hannan and Deistler, 1988). However, Moran proposed the limitations of linear model in the 1950s (Moran, 1953). The non-linear time-series model follows to become an attracting research topic until the late 1970s and early 1980s. These research studies include the threshold autoregressive model, exponential autoregressive model, bilinear model, non-linear autoregressive model, and state-dependent model. Tong et al. gave the threshold autoregressive model (Tong, 1983), and Ozaki proposed an exponential autoregressive model (Ozaki, 1980). The system identification is generally based on the complete clarity of input–output causality. In practical application, the system output can be measured, but the input of some specific systems is difficult to observe and measure. In that situation, it is not easy to determine the causal relationship between input and output. In that case, the traditional system identification method is difficult to apply. Although the system’s input cannot always be determined, it is certain that there is a relationship between some known parameters or data and the system output. These known parameters or data can directly or indirectly affect the system output. If the relevant data are also regarded as the system input, then the time-series model with exogenous input is determined. Tong analyzed the non-linear time series with exogenous input, established the relationship between non-linear time series and non-linear dynamic system (chaos), and studied the prediction based on non-linear time series (Tong, 1990).

In this paper, a kind of time series construction method on AHTPA-EC_50_ and its corresponding AAS is proposed firstly. We can find a lot of fractal characteristics from the two group time series. Then, the two groups of constructed series are modeled as two different NARX time-series models. Furthermore, two NARXNNs are used to perform the proposed model. And then we further proposed a prediction method for AHTPA-EC_50_ based on two NARXNNs and ML classification algorithms. The model and prediction method are useful and meaningful on antihypertensive active peptide research, drug design, and industrial production.

## 2 Materials and Methods

### 2.1 Analysis of AHTPA-EC_50_ and Its Corresponding AAS

#### 2.1.1 Statistical Analysis of AHTPA-EC_50_

**FIGURE 1:**
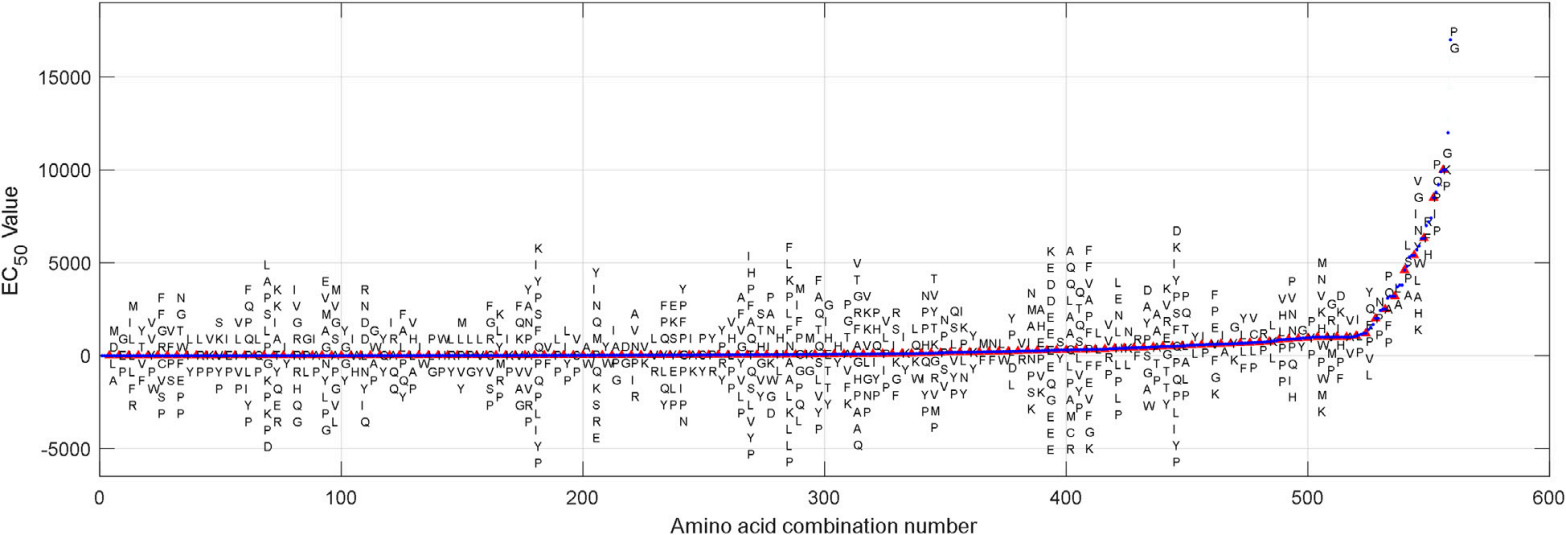
Constructed time series of natural logarithm of AHTPA-EC_50_ and its corresponding amino acid combination.

**FIGURE 2:**
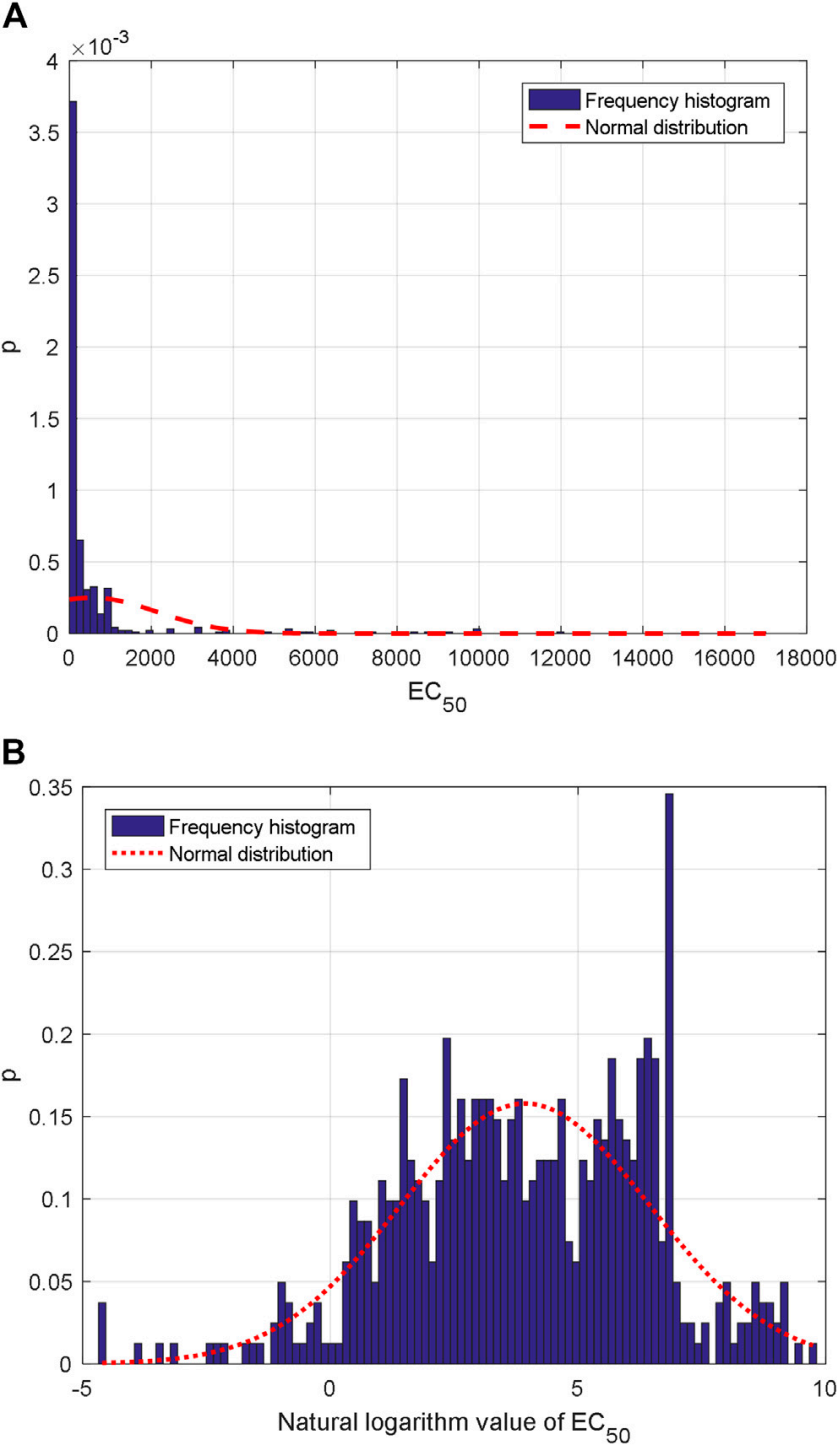
Statistical histogram: frequency histogram of **(A)** AHTPA-EC_50_ and **(B)** natural logarithm of AHTPA-EC_50_.

559 group AHTPA-EC_50_ data and their corresponding AAS are shown in Figure 1. Due to the difficulty of display, Supplementary Material marks the corresponding AAS every four EC_50_ values (interval = 3). The statistical histogram is analyzed, and histogram analysis of AHTPA-EC_50_ is shown in Figure 2A. We can see that AHTPA-EC_50_ is concentrated on the right side of the longitudinal axis of the coordinate and there is some very large AHTPA-EC_50_ value in these data. The characteristics of large distribution span and asymmetry appear in AHTPA-EC_50_ data. Comparing with the normal distribution data with the same mean and variance, it can be seen that AHTPA-EC_50_ data deviate very far from the normal distribution. In order to reduce the scale of AHTPA-EC_50_, the natural logarithm of AHTPA-EC_50_ data is calculated. The distribution of natural logarithm of AHTPA-EC_50_ is further analyzed, and the histogram distribution is shown in Figure 2B. Compared with the normal distribution of the same mean and variance, the natural logarithm histogram of AHTPA-EC_50_ cut off more slowly in the tail, and it shows the characteristics of a long tail. This is an important feature of fractal data.

#### 2.1.2 Encoding for AAS

The expression of amino acid is different from the digital number, and it is a symbolic quantity that cannot be directly quantified. In order to analyze the relationship between the AAS and its corresponding AHTPA-EC_50_, it is necessary to encode for the AAS. The numerical definitions of different amino acids are shown in Table 1. The AAS is digitally encoded in a 21 base system. Because the number 0 cannot appear in the first place of the combined code, the number 0 is not defined here.

**TABLE 1:** Numerical definitions of amino acids.

Amino acids	A	C	D	E	F	G	H	I	K	L
Numerical definitions	1	2	3	4	5	6	7	8	9	10
Amino acids	M	N	P	Q	R	S	T	V	W	Y
Numerical definitions	11	12	13	14	15	16	17	18	19	20

##### 2.1.3 Constructed Time Series and Its Time–Frequency Characteristics

(1) Constructed time series based on sorting code of AAS

**FIGURE 3:**
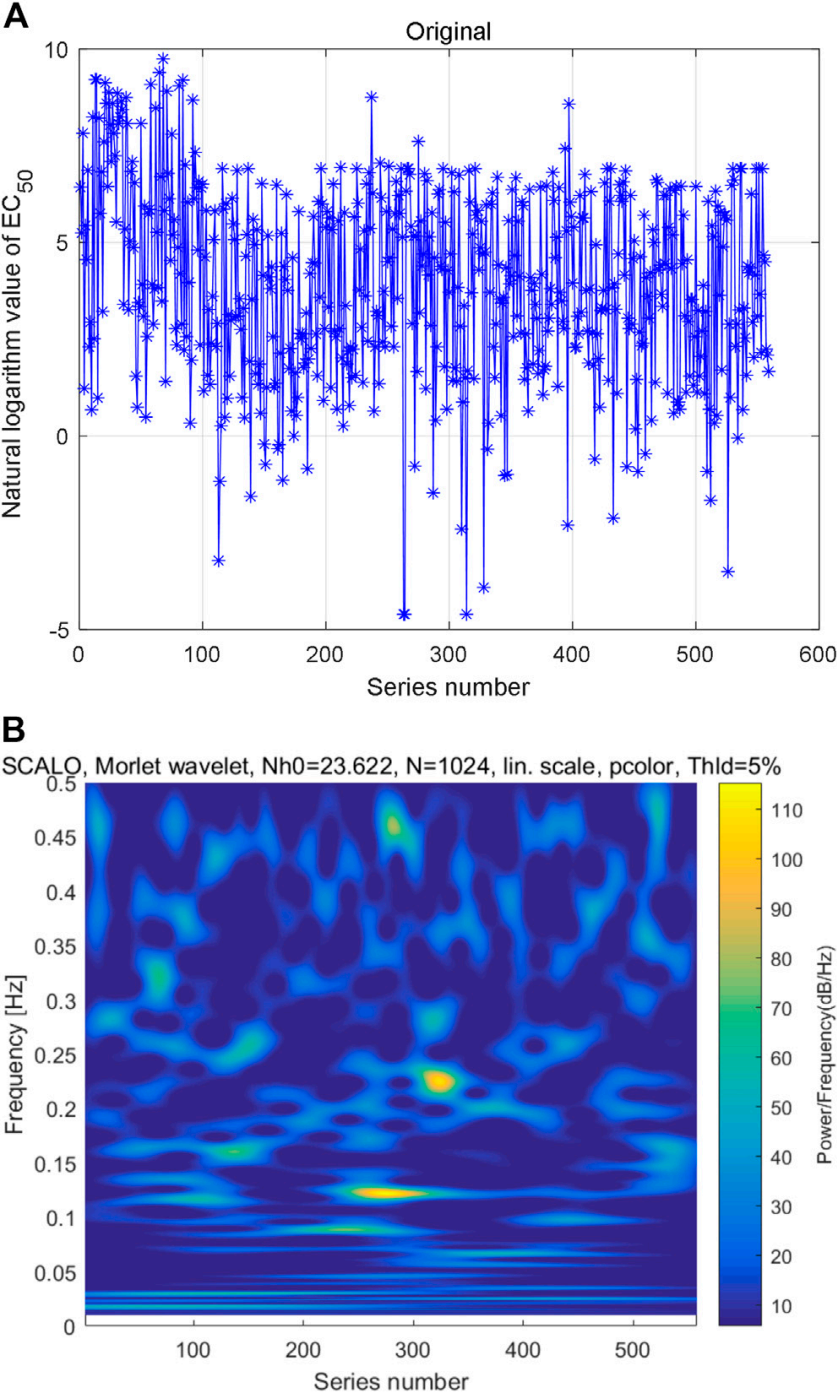
Constructed first time series and its multi-scale wavelet transform: **(A)** time series of natural logarithm of AHTPA-EC_50_ and **(B)** time–frequency distribution of multi-scale wavelet transform.

As mentioned above, the AAS can be converted to decimal digit by numerical definitions of amino acids. After sorting the natural logarithm of coding numbers from small to large, the natural logarithm of AHTPA-EC_50_ can be constructed. The constructed time series is shown in Figure 3A. Multi-scale wavelet transform is performed to the constructed AHTPA-EC_50_ time series, and the time–frequency distribution is shown in Figure 3B. There is also no obvious law between high-energy data and series number and frequency in Figure 3B, and different time–frequency relationships show similar patterns.

(2) Constructed time series based on sorting AHTPA-EC_50_

**FIGURE 4:**
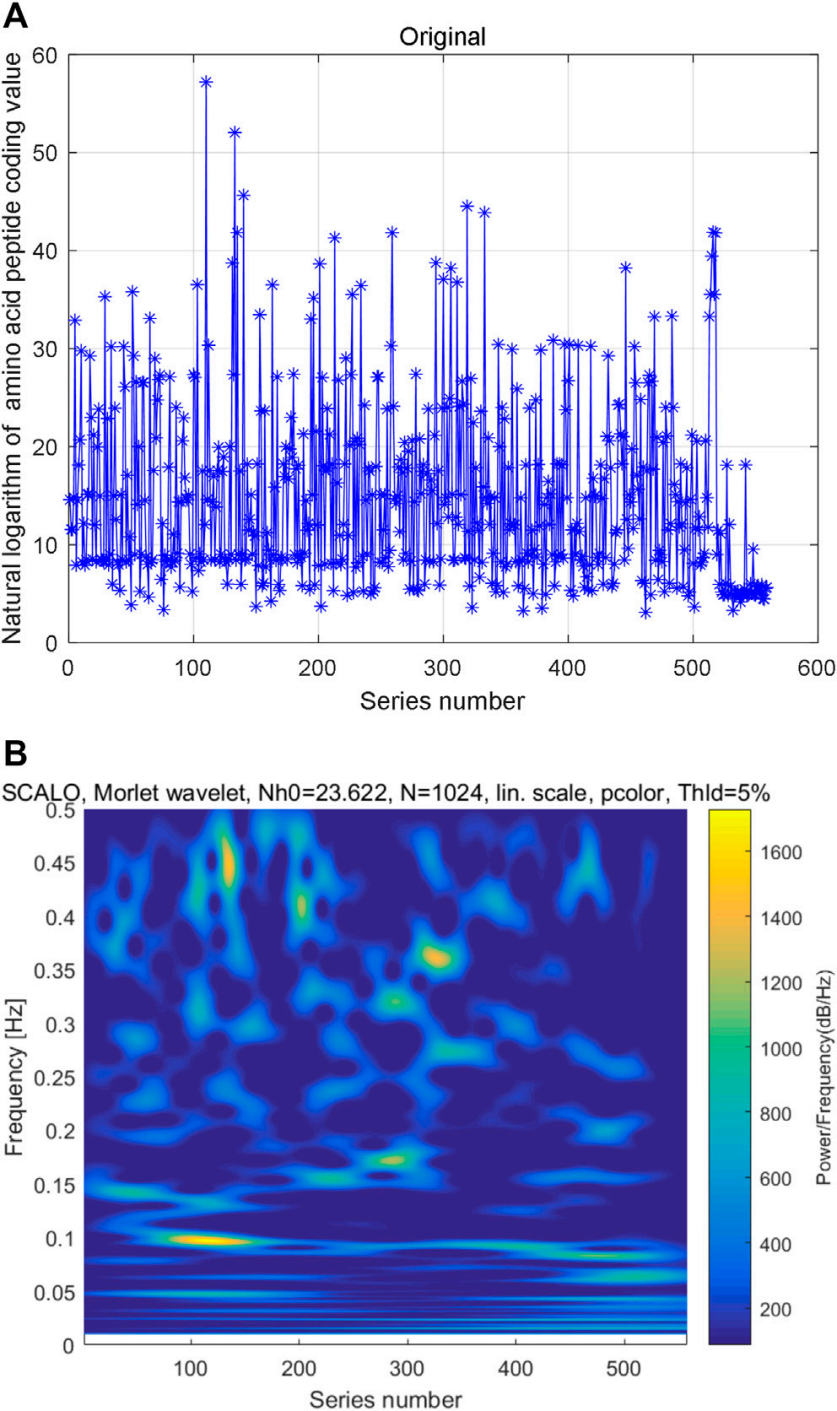
Constructed second time series and its multi-scale wavelet transform: **(A)** time series of natural logarithm of coding AAS and **(B)** time–frequency distribution of multi-scale wavelet transform.

We also constructed natural logarithm of AHTPA-EC_50_ time series by sorting the data from small to large. The AAS is converted to decimal digit by numerical definitions of amino acids. After sorting the natural logarithm of AHTPA-EC_50_ from small to large, the time series of natural logarithm of coding value of AAS is also constructed. The constructed time series is shown in Figure 4A. Multi-scale wavelet transform is performed to the natural logarithm of coding value of AAS. The constructed time series of AAS and its time–frequency distribution are shown in Figure 4B. And there is no obvious law between high-energy data and series number and frequency. However, different time–frequency relationships show similar patterns.

In summary, the relationship between the natural logarithm of AHTPA-EC_50_ and its corresponding natural logarithm of coding AAS is special. If one of the series is sorted, the other will be a random number series. We deduce that there is not a direct regression modeling for their relationship.

**FIGURE 5:**
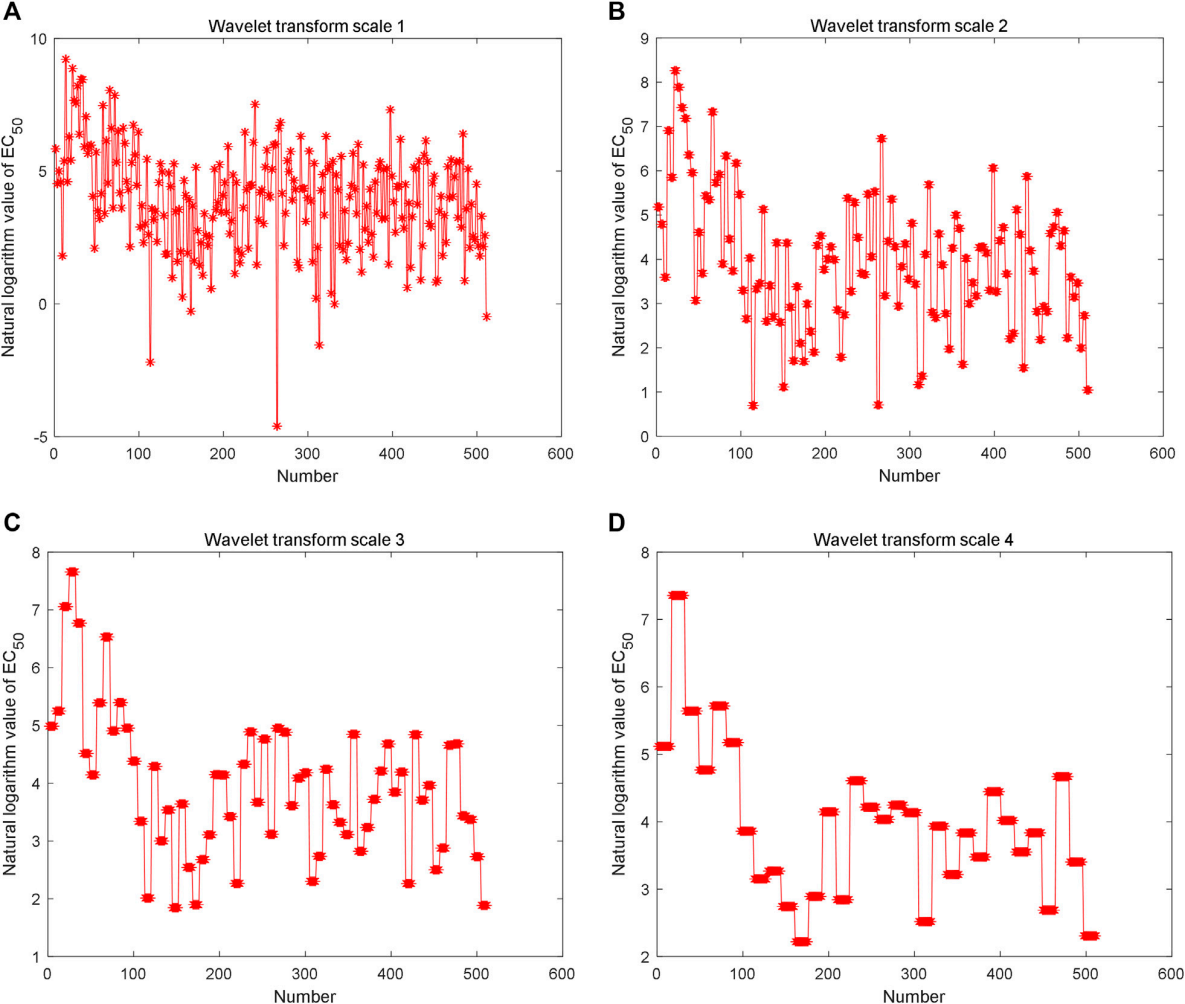
Multi-scale wavelet decomposition of constructed time series: low frequency data of **(A)** level 1 wavelet transform, **(B)** level 2 wavelet transform, **(C)** level 3 wavelet transform, and **(D)** level 4 wavelet transform.

**FIGURE 6:**
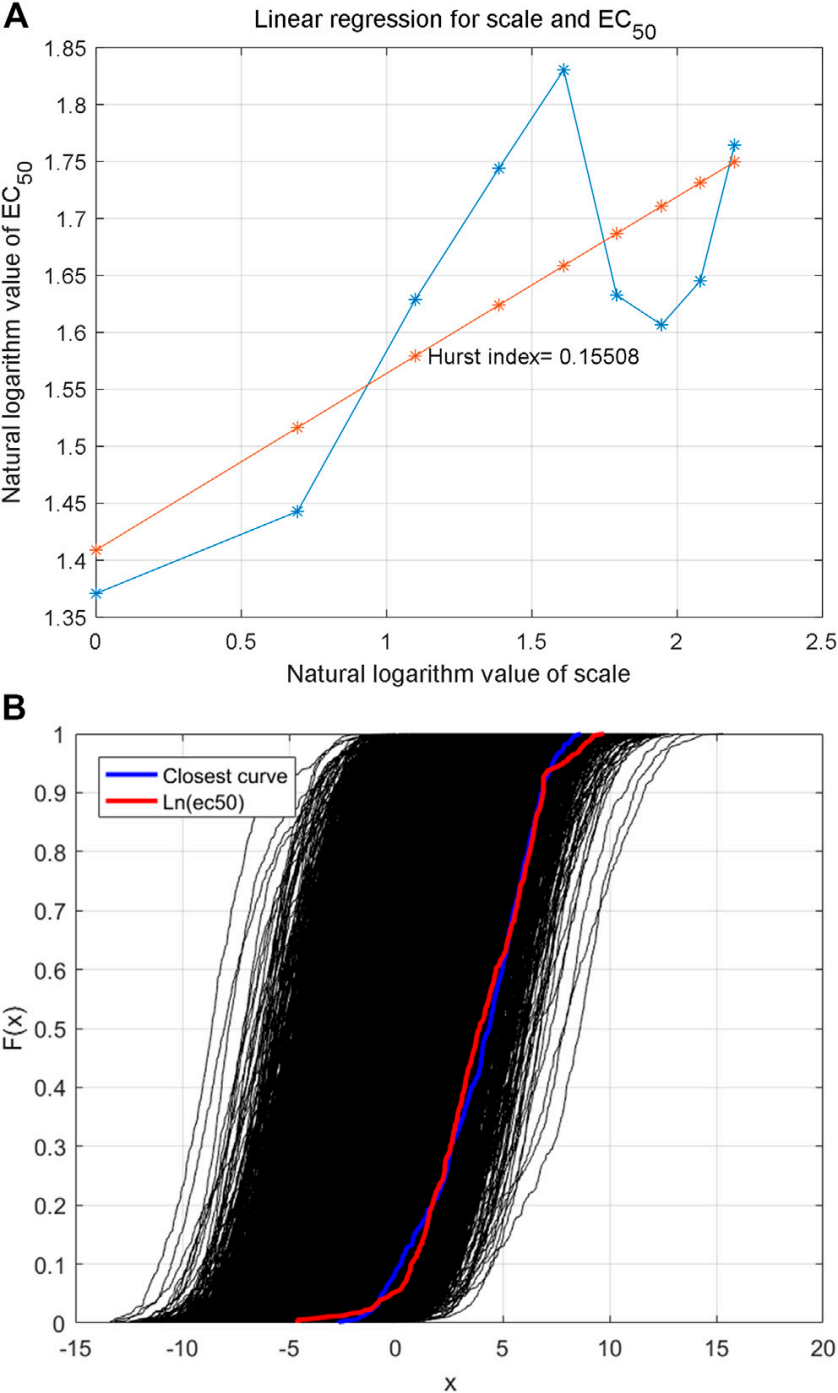
Estimation of Hurst index of the time series **(A)** and empirical probability distribution of FBM with the same Hurst index **(B)**.

The Haar wavelet is further used to decompose the reconstructed time series to analyze fractal characteristics (data in Figure 3A) in multiple scales. The low-frequency data of different scales are shown in Figures 5A,B,C,D. The Hurst index of the time series is estimated by multi-scale wavelet transform data, as shown in Figure 6A, in which the wavelet transform scales are 1–9. The estimated Hurst index is used to generate FBM, and the empirical probability distribution of the generated FBM data is shown in Figure 6B. 10,000 FBM data are generated by the Monte Carlo method here. The probability distribution data corresponding to the constructed natural logarithm of AHTPA-EC_50_ are represented in red, and the curve closest to the constructed natural logarithm of AHTPA-EC_50_ is shown in blue. It can be seen that the constructed AHTPA-EC_50_ is very close to the FBM time series.

#### 2.2 Non-Linear Autoregressive Time-Series Modeling and Its Implementation

##### 2.1.4 Correlation Analysis

**FIGURE 7:**
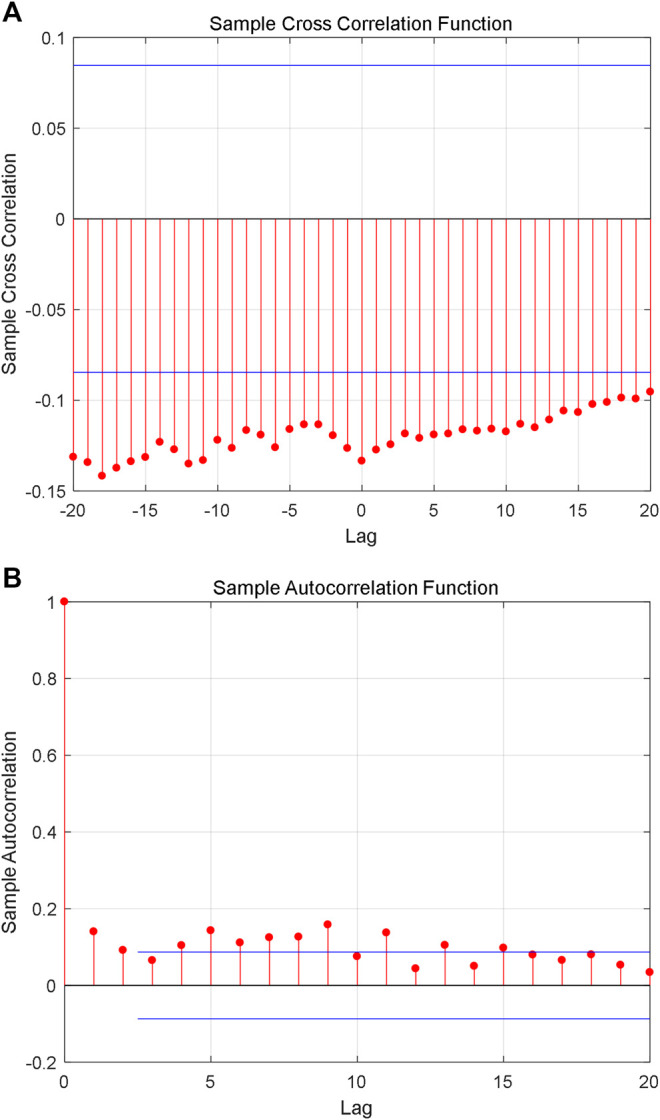
Correlation analysis of the second group time series. **(A)** Cross-correlation with the sorting natural logarithm of coding AAS. **(B)** Autocorrelation of natural logarithm of AHTPA-EC_50_.

**FIGURE 8:**
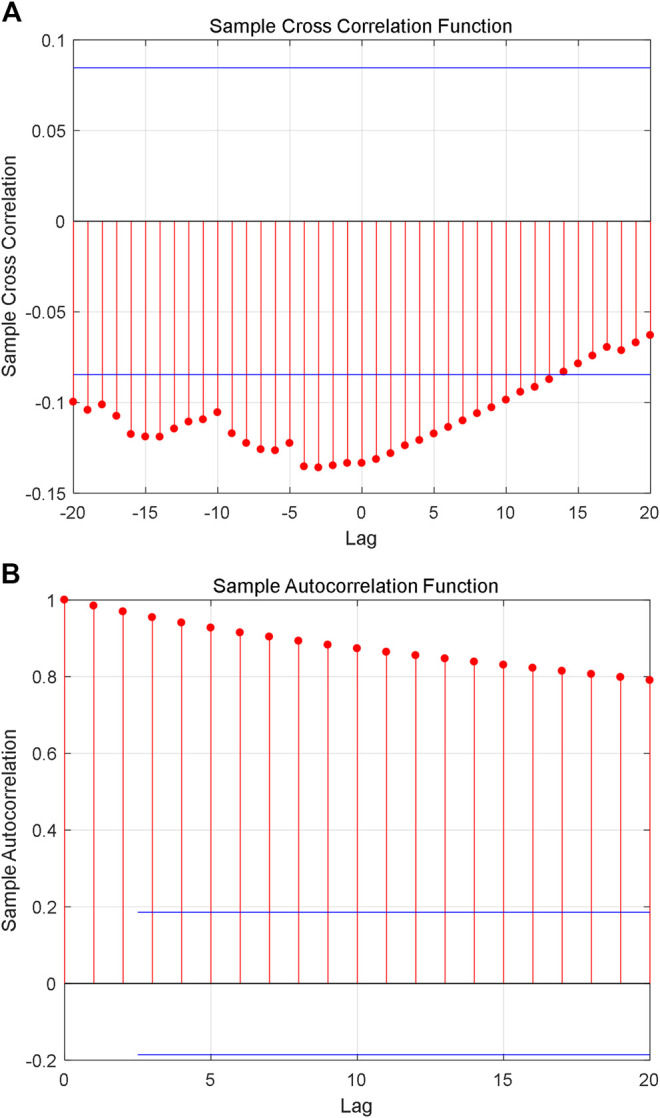
Correlation analysis of the first group time series. **(A)** Cross-correlation with the natural logarithm of coding value of AAS. **(B)** Autocorrelation of sorting natural logarithm of AHTPA-EC_50_.

Although the constructed series shows fractal characteristics, the relationship between the natural logarithm of coding value of AAS and its corresponding natural logarithm of AHTPA-EC_50_ still needs to be analyzed. Figure 7A shows the cross-correlation analysis for the first group of constructed time series, and it shows weak correlation between the two time series. Figure 7B shows the autocorrelation analysis for sorting natural logarithm of AHTPA-EC_50_. We can see that the sorting natural logarithm of AHTPA-EC_50_ showed weak autocorrelation. Figure 8A shows the cross-correlation analysis for the second group of time series, and it shows weak correlation between the two time series. Figure 8B shows the autocorrelation analysis for constructed natural logarithm of AHTPA-EC_50_, and the natural logarithm of AHTPA-EC_50_ based on the coding value AAS showed obvious autocorrelation.

##### 2.1.5 Non-Linear Autoregressive Model With Exogenous Input

According to the above analysis, the two groups’ constructed AHTPA-EC_50_ data are modeled as an autoregressive time series, and the natural logarithm of coding AAS is used as the exogenous input parameter. The non-linear autoregressive model with exogenous input is established to describe the relationship between the AAS and its corresponding AHTPA-EC_50_, and this relationship is described as

$$y\left(t\right)=f[\begin{array}{l} y\left(t-1\right),y\left(t-2\right),...,y\left(t-n_{y}\right)\\ u\left(t-1\right),u\left(t-2\right),...,u\left(t-n_{u}\right) \end{array}],$$ (1)

where $y\left(t\right),y\left(t-1\right),y\left(t-2\right),...,y\left(t-n_{y}\right)$ represent time series at different time and $u\left(t-1\right),u\left(t-2\right),...,u\left(t-n_{u}\right)$ represent exogenous inputs at different time, y denotes the natural logarithm of AHTPA-EC_50_, and u denotes the natural logarithm of coding AAS value. According to the characteristics of AAS and its corresponding AHTPA-EC_50_, the AAS is defined as the input parameter affecting AHTPA-EC_50_ here.

##### 2.1.6 Neural Network Implementation of Model

**FIGURE 9:**
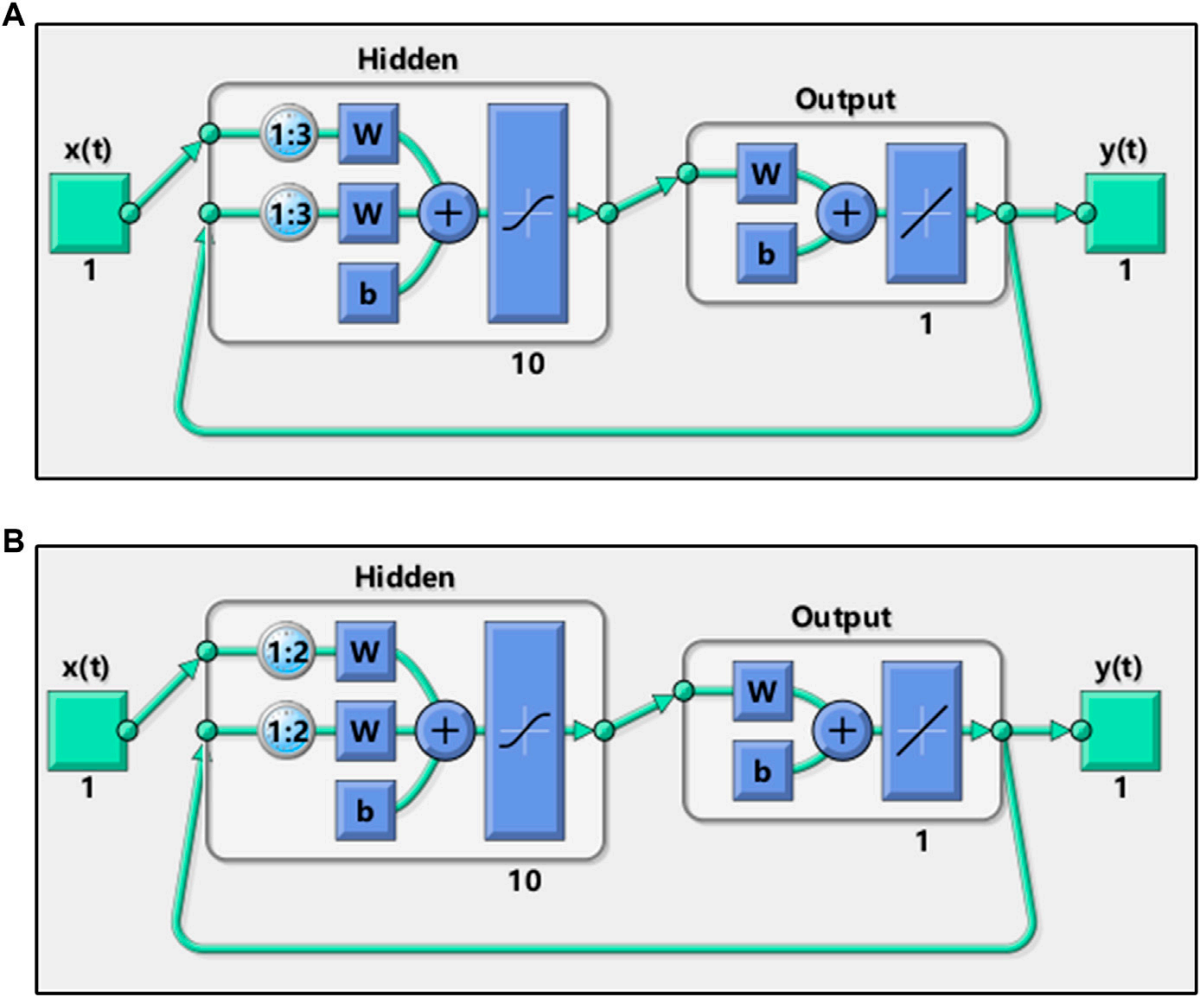
Structures of the neural network for the **(A)** first series and **(B)** second series.

The NARX model of AHTPA-EC_50_ and AAS was realized by the NARXNN. This neural network was performed in Matlab. The two neural network structure**s** are shown in Figure 9. The mean square error (MSE) is selected as the performance function of NARXNN. The Levenberg–Marquardt algorithm is used for net training. The division ratio of training set, verification set, and test set in neural network learning samples is 0.7:0.15:0.15. The delay corresponding to the two constructed series is 1:3 and 1:2, respectively, and the hidden layer has 10 neurons.

##### 2.1.7 Prediction Method for AHTPA-EC_50_

**FIGURE 10:**
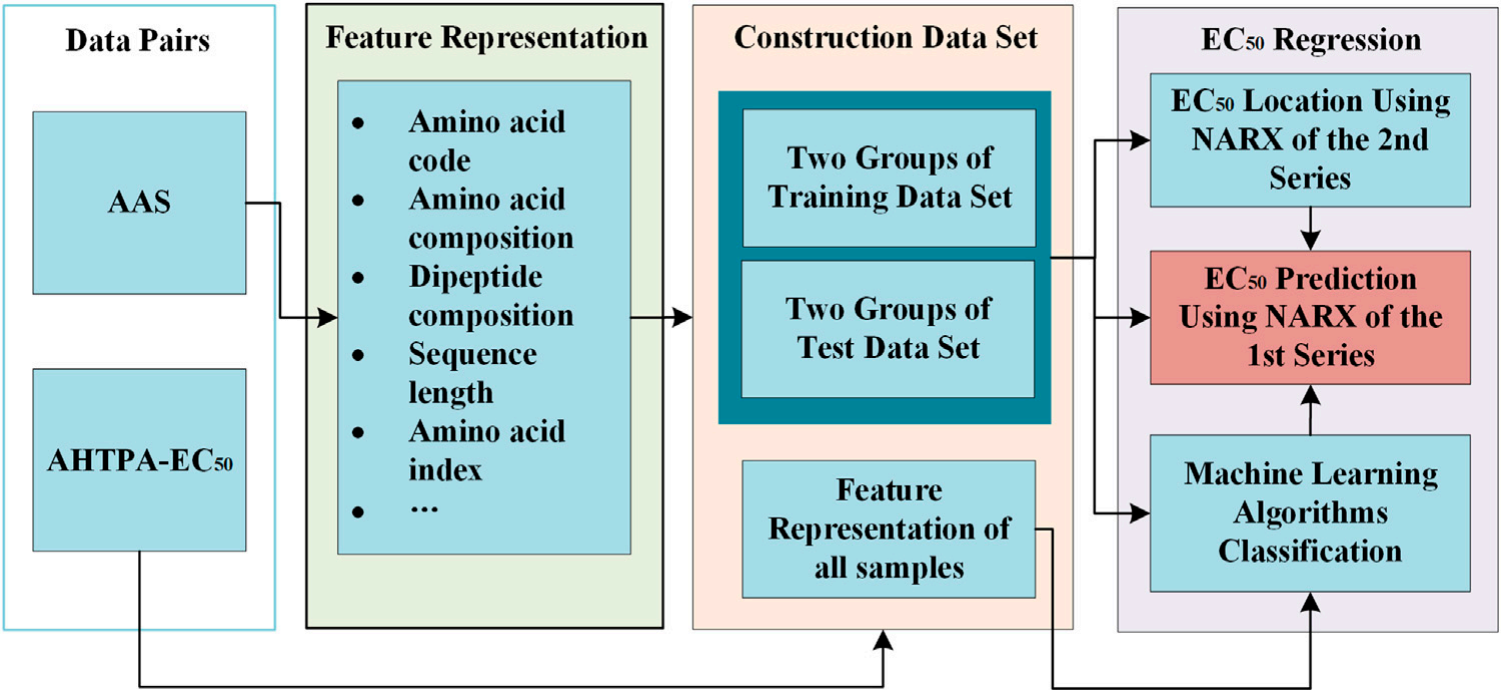
Prediction method for AHTPA-EC_50_–based NARX.

We further proposed a method for AHTPA-EC_50_ prediction. This method includes two parts: classification and AHTPA-EC_50_ prediction. The ML algorithm is used to classify the AAS. The classification corresponds to different digital segments of AHTPA-EC_50._ The feature representation is necessary in this process. This prediction method is described in Figure 10. Support vector machine (SVM) is used for classification in this research.

### 3 Results

#### 3.1 Prediction Results of the Proposed Model

**FIGURE 11:**
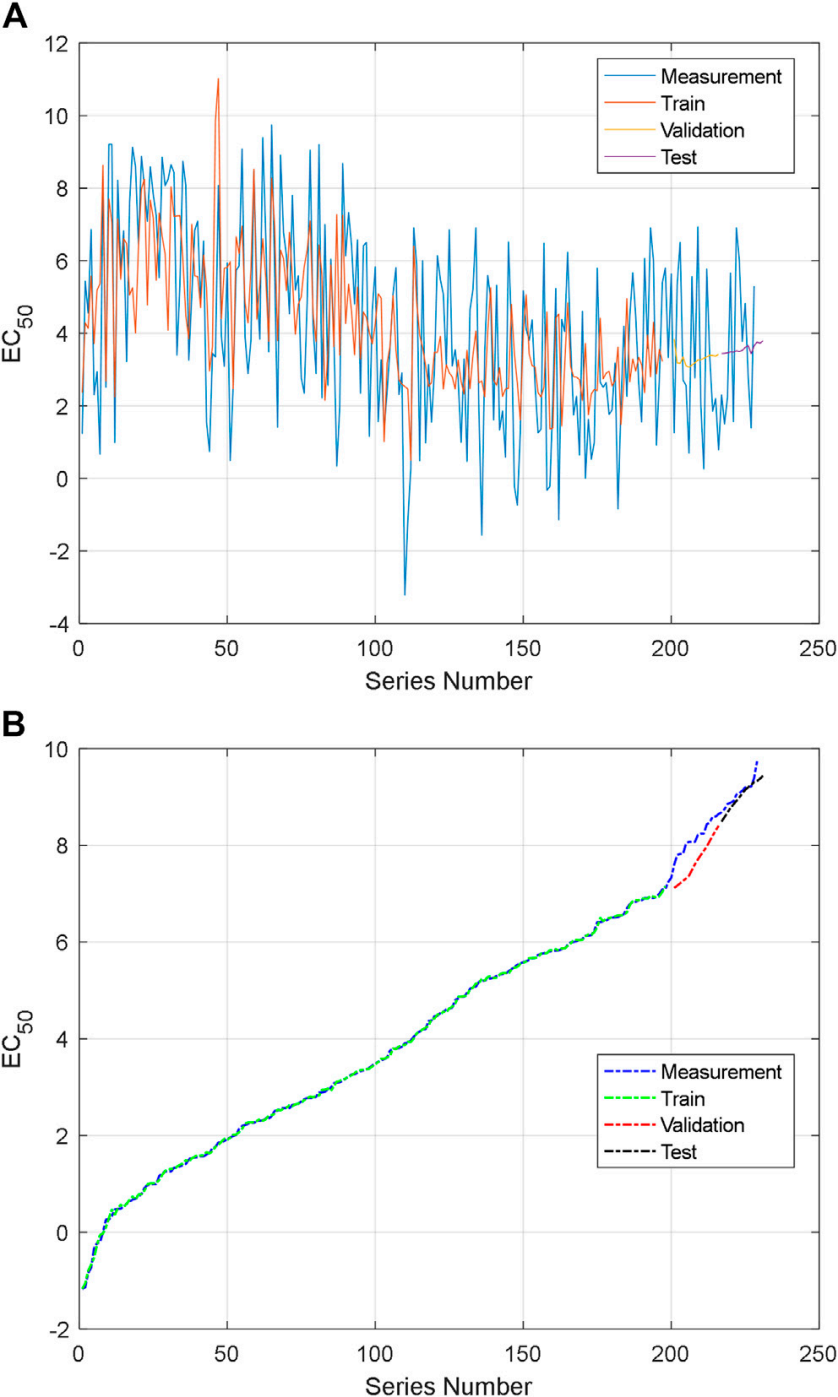
Training and testing data: **(A)** the first NARXNN prediction for the first group series and **(B)** the second NARXNN prediction for the second group series.

As mentioned above, there are 559 groups of samples in total. However, these data include different antihypertensive peptides, whose length is from 2 to 20. We select the samples of AAS, whose length is fixed. There are 231 samples of dipeptides and tripeptides in our dataset. They are larger than other peptides. These data are used to verify the proposed model and prediction model. We also constructed two series according to the above method. The first 200 groups in the first series of samples are used for training, and the last 31 data are used for validation and testing. The training results of the constructed series are shown in Figure 11.

For the first NARXNN corresponding to the first group series, the training error is 4.895193, the validation error is 4.636605, and the testing error is 3.546904. For the second NARXNN corresponding to the second group series, the training error is 0.001881, the validation error is 0.124045, and the testing error is 0.010165. The second NARXNN has high accuracy; however, it needs the sorting number, and it cannot be used for prediction alone. The classification of the proposed prediction method can provide a rough location in the series. The first NARXNN also gives an original estimation value of AHTPA-EC_50_. The AHTPA-EC_50_ will be predicted in the segment of the second series, and two known term AASs help in prediction. The known AASs are selected by the rough location and original estimation value. The second NARXNN is trained every time; therefore, the output will be changed slightly. The first and second NARXNNs are trained in Figures 11A,B.

**FIGURE 12:**
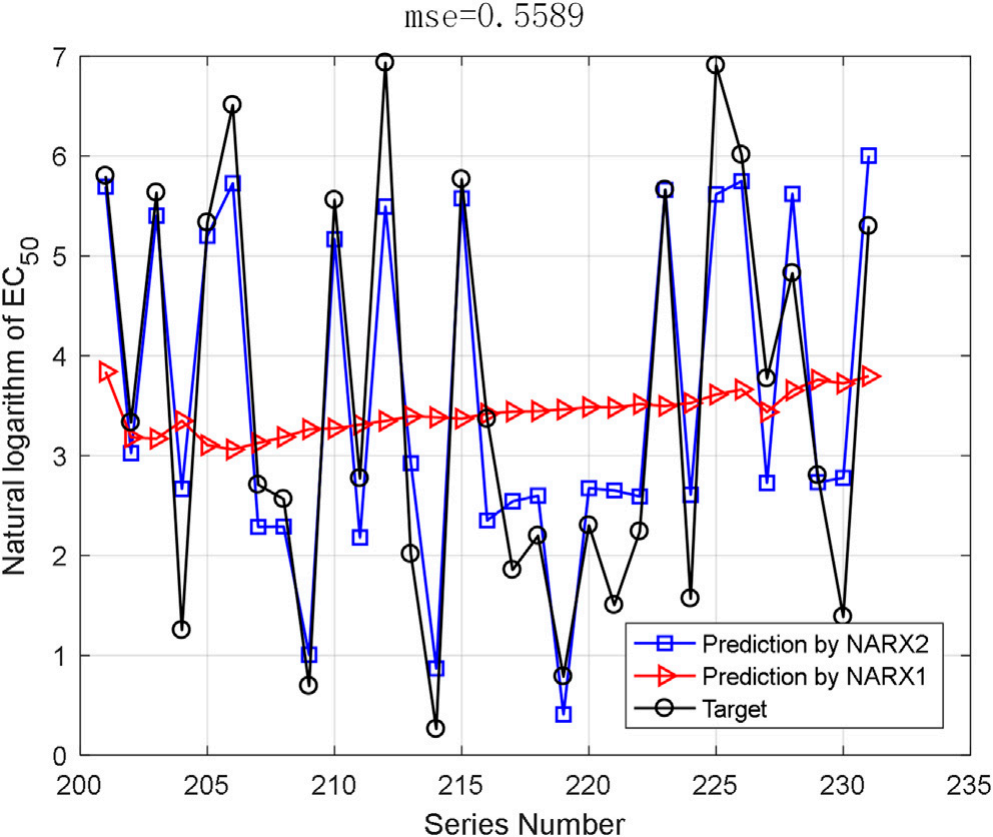
Prediction results by the proposed method.

**FIGURE 13:**
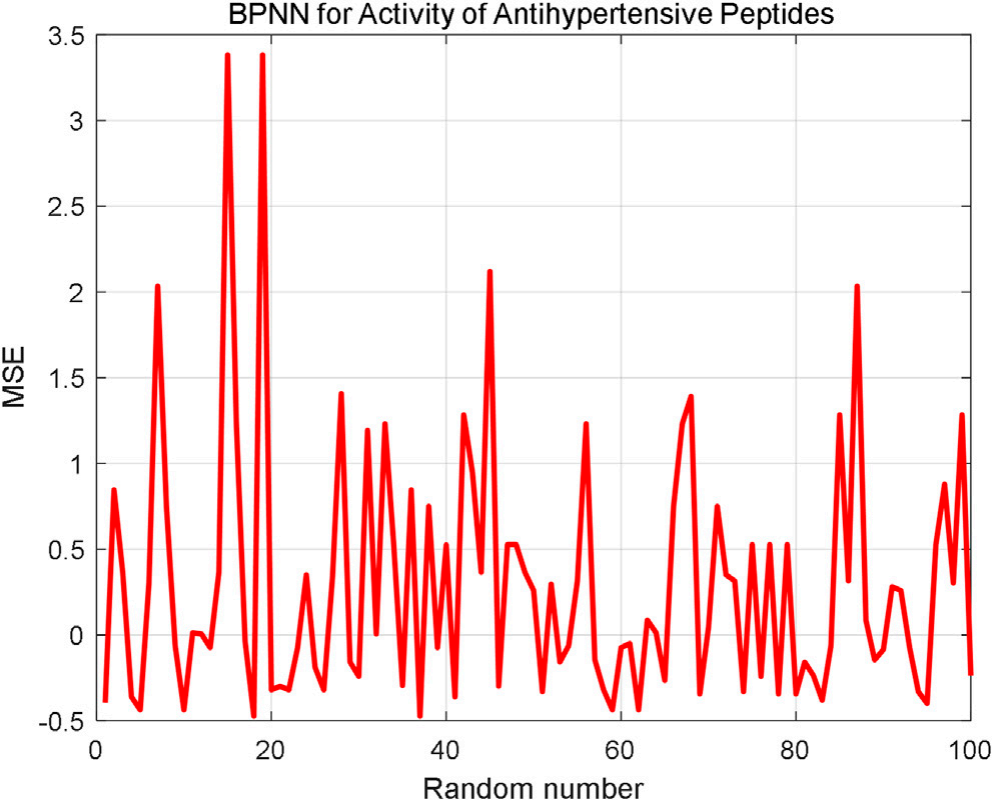
Prediction results by the BPNN.

The AHTPA-EC_50_ of dipeptides and tripeptides is used to verify the prediction method. The first 200 groups in the first series of samples are used for training, and the last 31 data in the first series are used for testing. The proposed method demands classification, and we assume that the classification is correct here; thus, we input the AAS in segments. And the classification is designed as three classification. AHTPA-EC_50_ = 1, and median values of the series are designed as segment points. The results of prediction are shown in Figure 12. Therefore, when the classification is correct, the MSE is 0.5589. We also designed a backpropagation neural network (BPNN) for comparison. The network structure is designed as 3–10–1. The mean square error (MSE) is selected as a performance function. The Levenberg–Marquardt algorithm is used for net training. The logsig function is set as the input function, and the pure linear function is used in the second layer. The number of iterations is set to 1000, the learning rate is 0.1, and the learning target is 0.00001. The results are shown in Figure 13, where test samples are randomly selected 100 times. The results reveal that the proposed method has better accuracy than the BPNN.

#### 3.2 Classification of AAS for AHTPA-EC_50_

**FIGURE 14:**
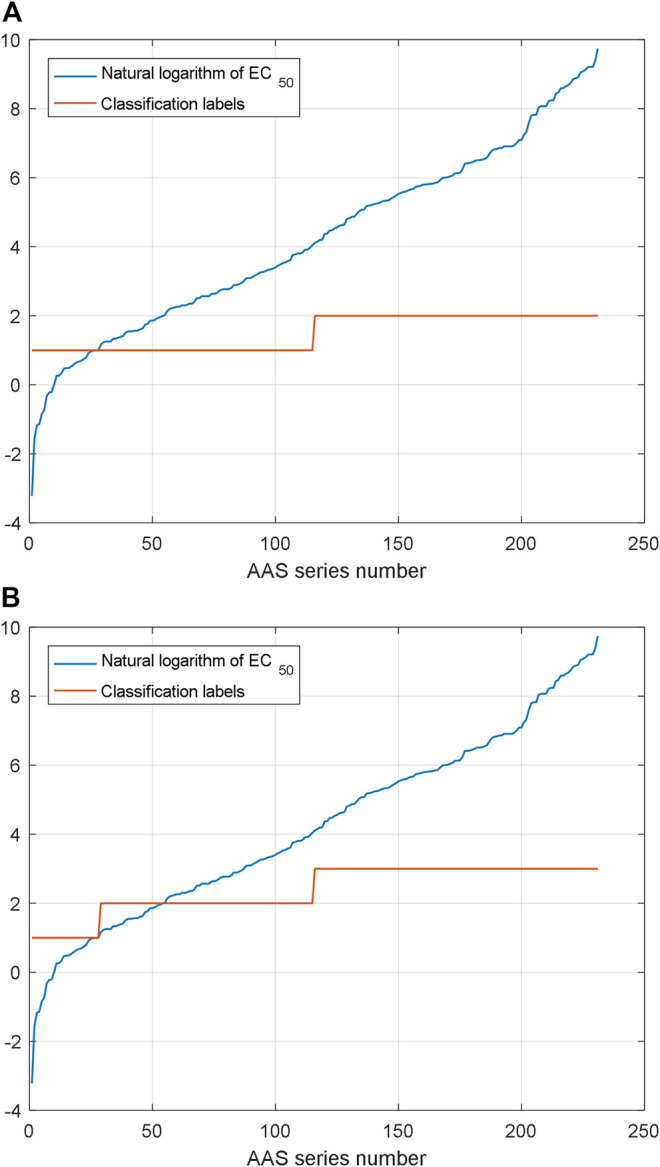
Classification by label design: **(A)** two classification and **(B)** three classification.

As mentioned above, the proposed prediction method demands a rough position which is used in NARX2 prediction. Two classification and three classification are designed for the proposed prediction method here. SVM is used for the classification of AHTPA-EC_50_ and its corresponding AAS here. We classify the AAS whose length is less than three amino acids. 231 samples of dipeptides and tripeptides are classified here. For three classification, AHTPA-EC_50_ = 1 and median values of the series are designed as segment points. For two classification, the median value of the series is designed as the segment point. The label design is shown in Figure 14.

**FIGURE 15:**
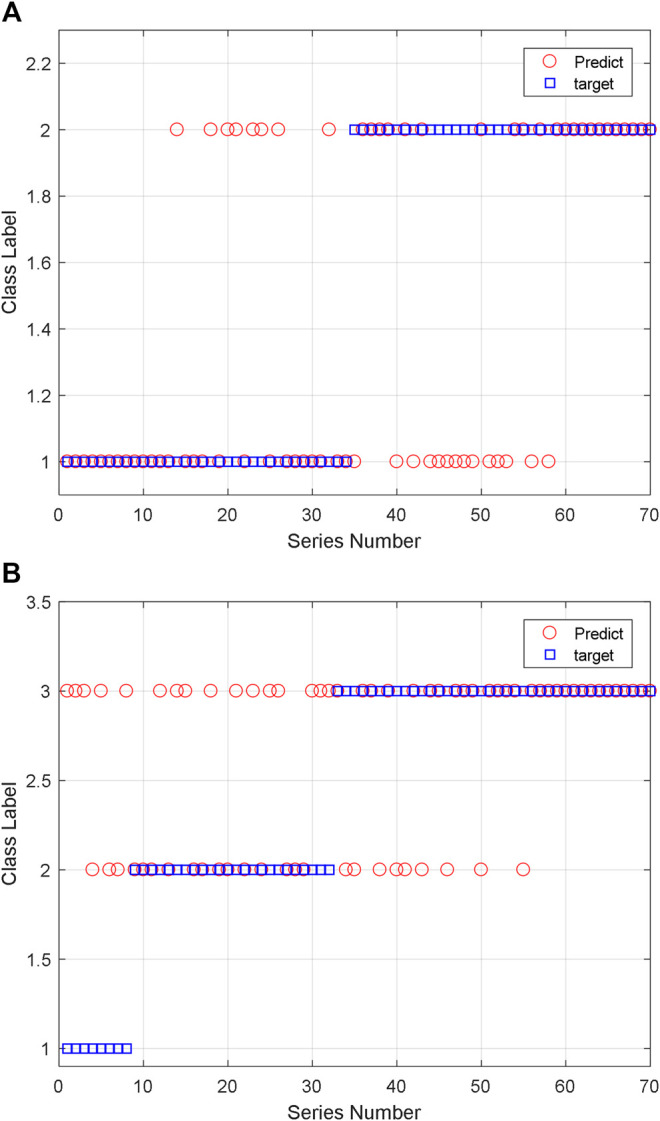
Classification results of the AAS using SVM: **(A)** two classification and **(B)** three classification.

For two classification, there are 161 training data pairs and 70 testing data pairs which are used for classification. And eight feature descriptors are extracted from the peptide sequence. They are the amino acid composition, the digital description of AAS, the peptide sequence code, and the length of peptide sequence. The classification results are shown in Figure 15. We can see that the two classification accuracy is 68.57% and the three classification accuracy is 60.00%. Due to the limitations in training, the effect of three classification is not very good. If the quantity of training sample increases and other ML algorithms are also used, we think the accuracy can be improved.

### 4 Conclusion

In this paper, the statistical distribution of AHTPA-EC_50_ is analyzed. Two group time series are constructed between AHTPA-EC_50_ and its corresponding AAS. According to the characteristics of constructed time series, AHTPA-EC_50_ is modeled by the NARX model. Then, a prediction method of AHTPA-EC_50_ is proposed. Dipeptides and tripeptides are used to verify the proposed model and prediction method. The results show that the MSE is 0.5589 when the classification is correct. Finally, we tried to classify the dipeptide and tripeptide data by SVM. Although the accuracy of classification is not very high, it is still feasible. The proposed model and prediction method provide a solution for AHTPA-EC_50_ prediction, and they are useful and meaningful on antihypertensive active peptide research, drug design, and industrial production (Chen et al., 2020; Granger and Joyeux 1980).

## Data Availability

The original contributions presented in the study are included in the article/Supplementary Material, and further inquiries can be directed to the corresponding author.
