# Supplementary figures and images for: Autoregressive Modeling and Prediction of the Activity of Antihypertensive Peptides
Source: Front Genet. 2022 Jan 11;12:801728. doi: 10.3389/fgene.2021.801728 (PMC8787326; doi:10.3389/fgene.2021.801728)

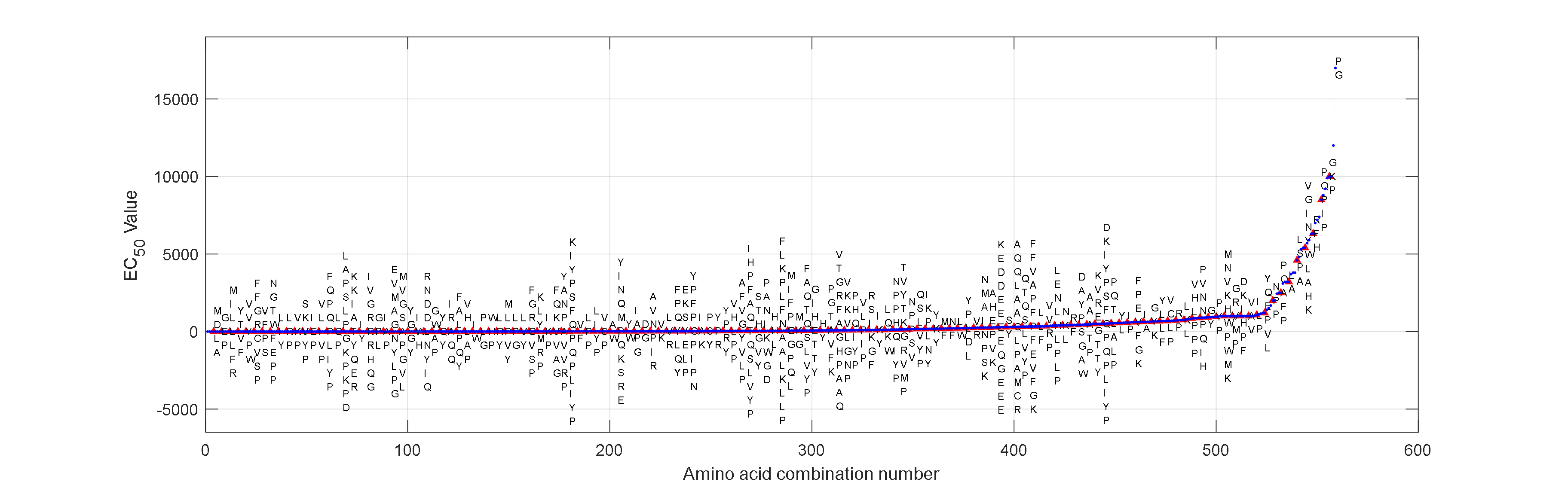

Supplement: Supplementary file 2 [file DataSheet1.zip › figures/fig1.tif]

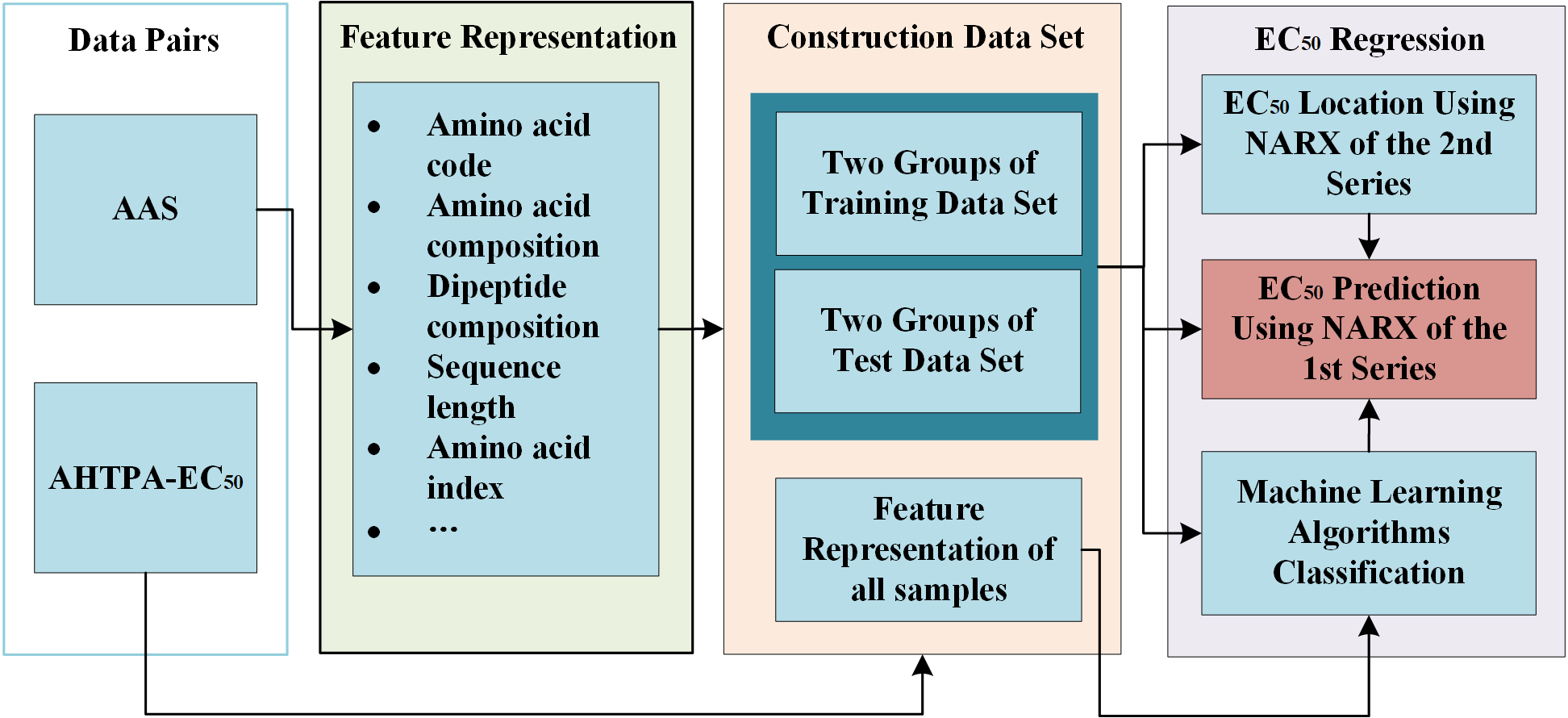

Supplement: Supplementary file 2 [file DataSheet1.zip › figures/fig10.tif]

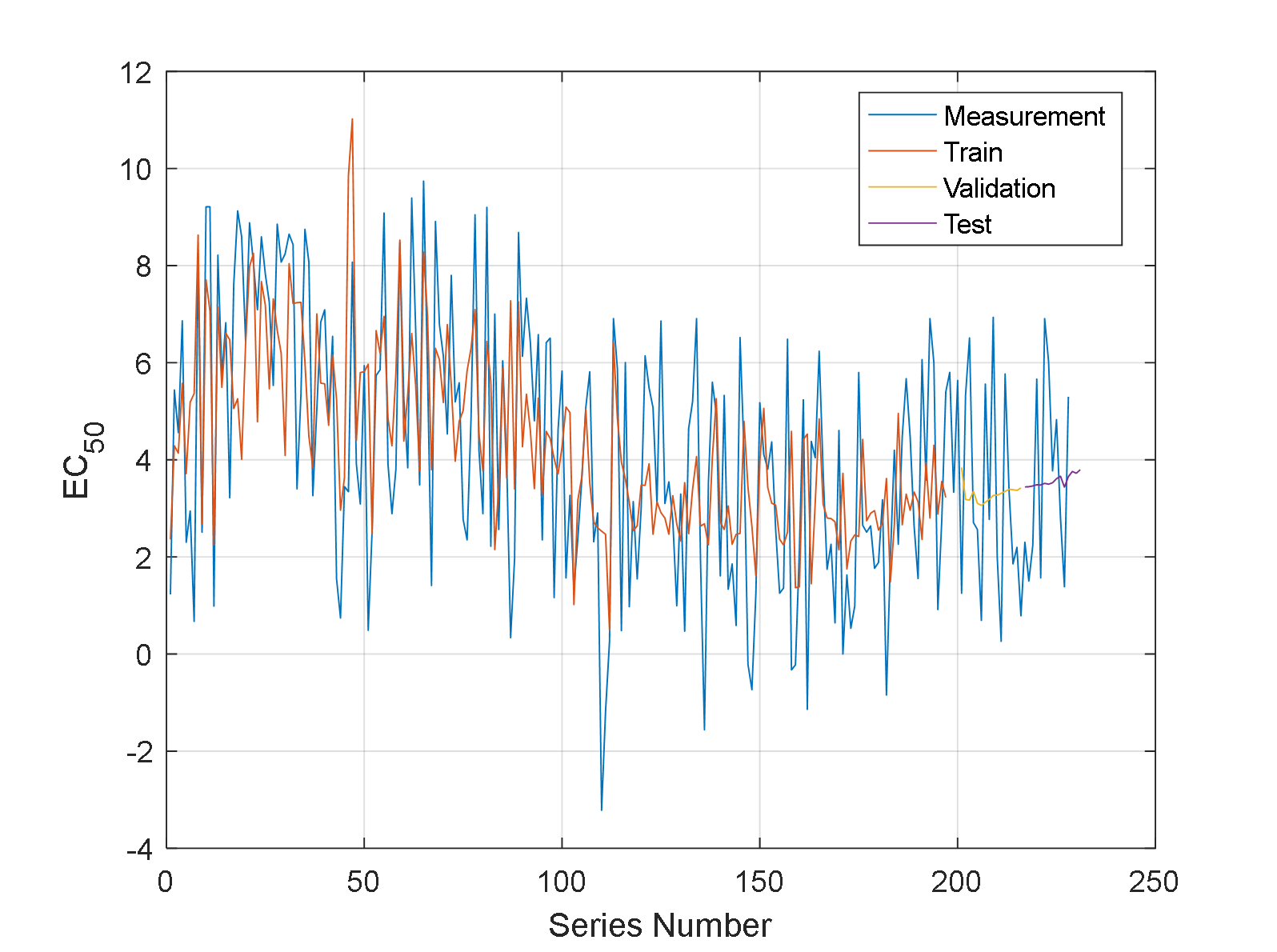

Supplement: Supplementary file 2 [file DataSheet1.zip › figures/fig11a.tif]

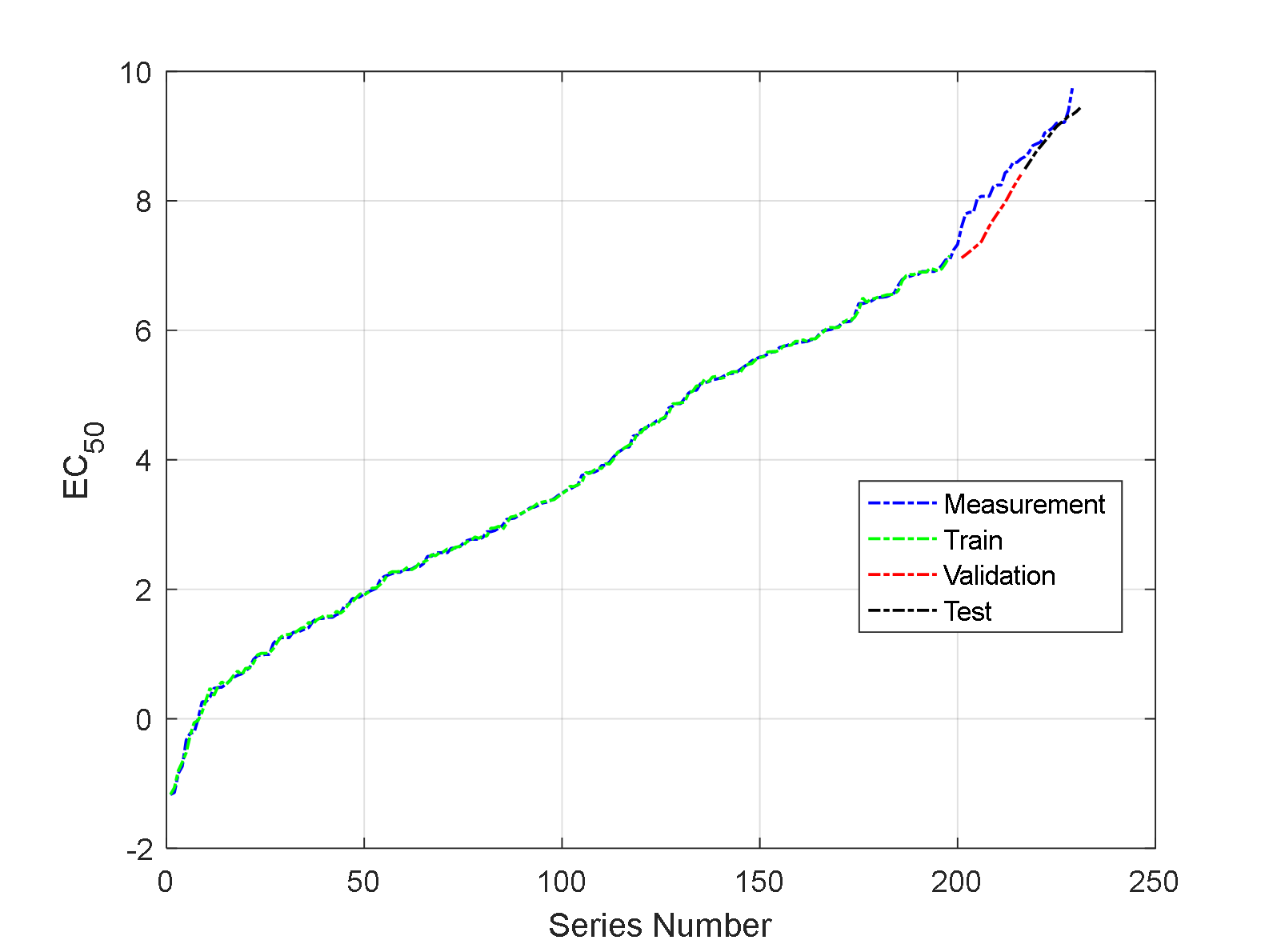

Supplement: Supplementary file 2 [file DataSheet1.zip › figures/fig11b.tif]

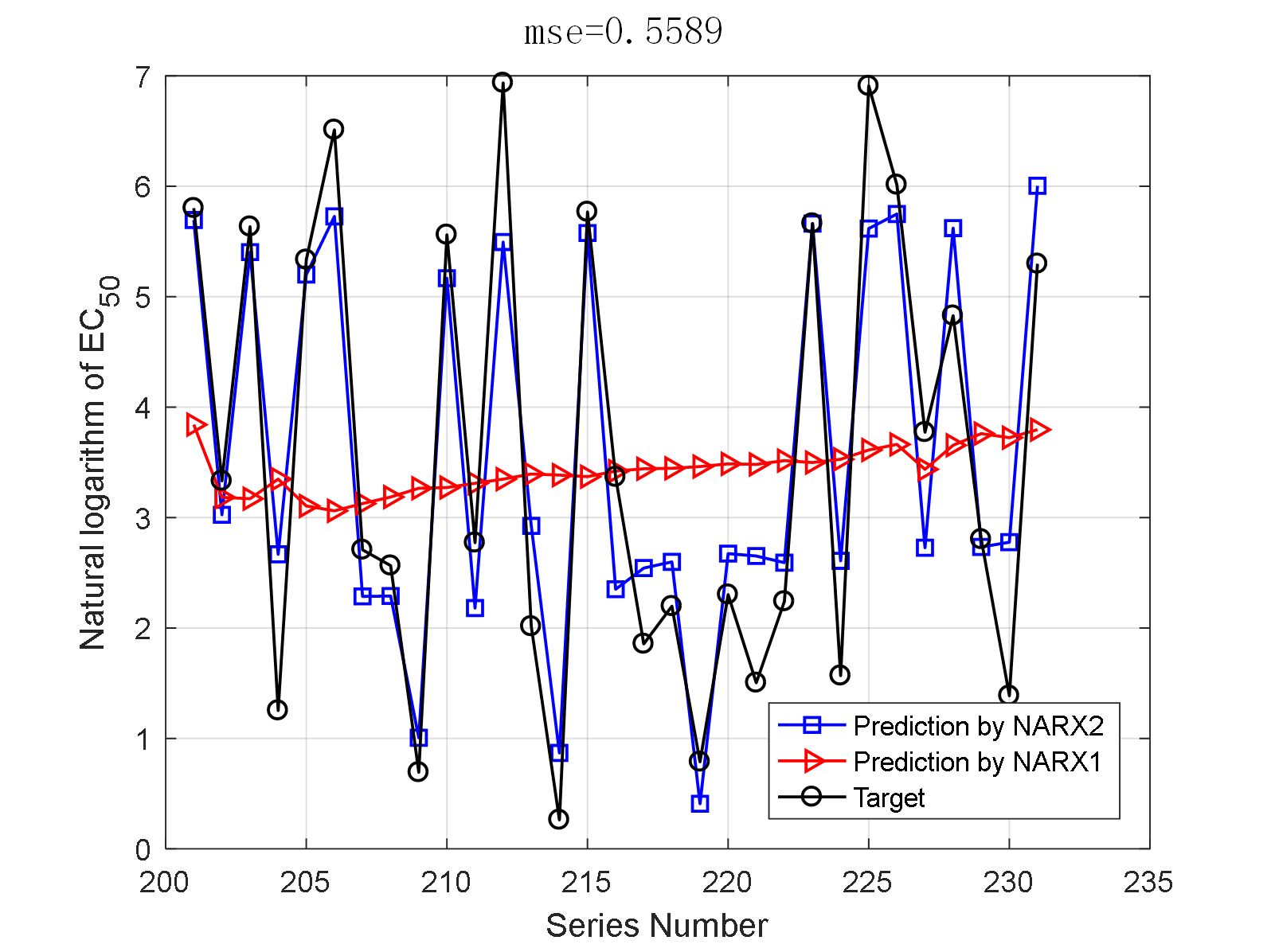

Supplement: Supplementary file 2 [file DataSheet1.zip › figures/fig12.tif]

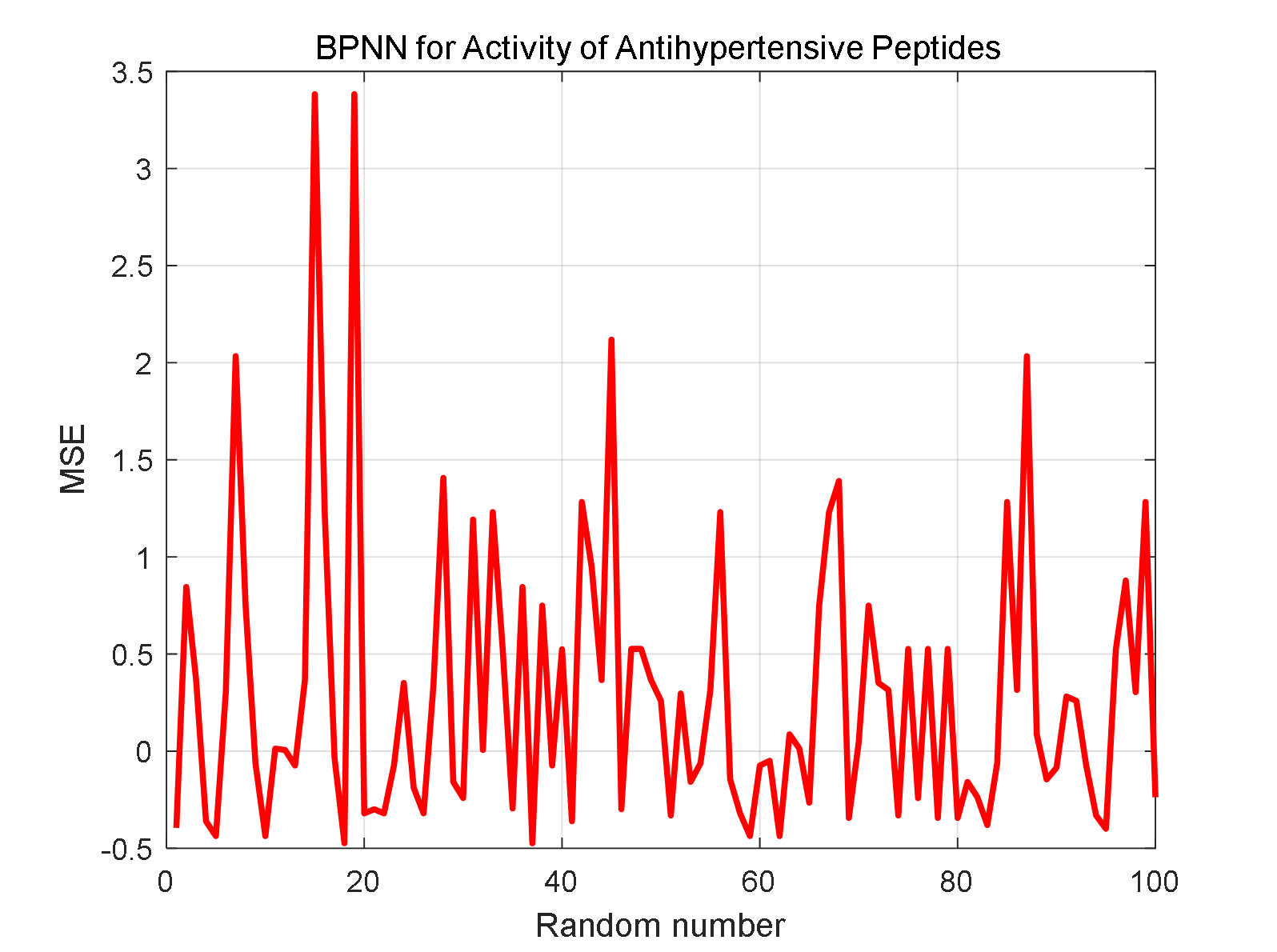

Supplement: Supplementary file 2 [file DataSheet1.zip › figures/fig13.tif]

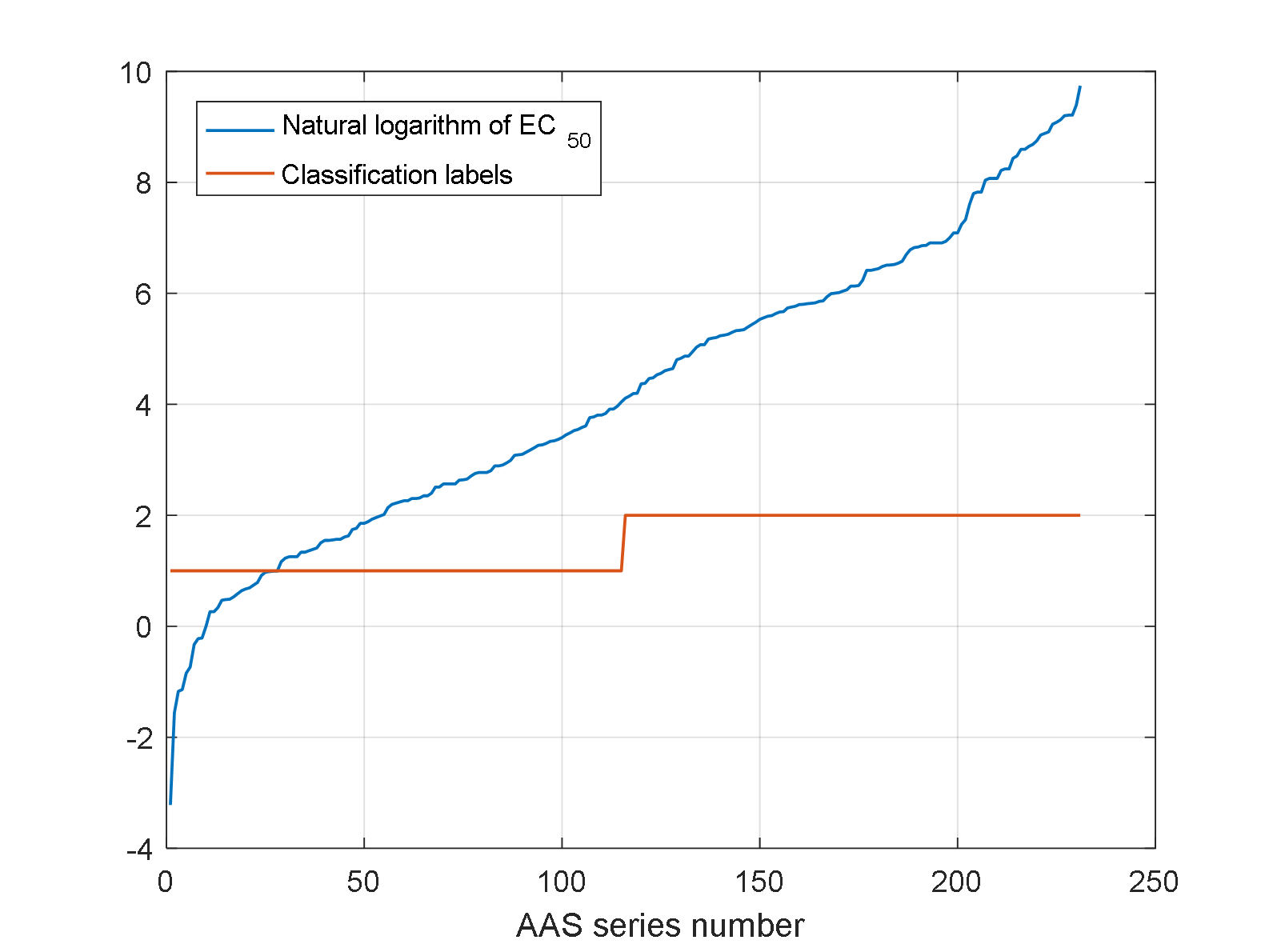

Supplement: Supplementary file 2 [file DataSheet1.zip › figures/fig14a.tif]

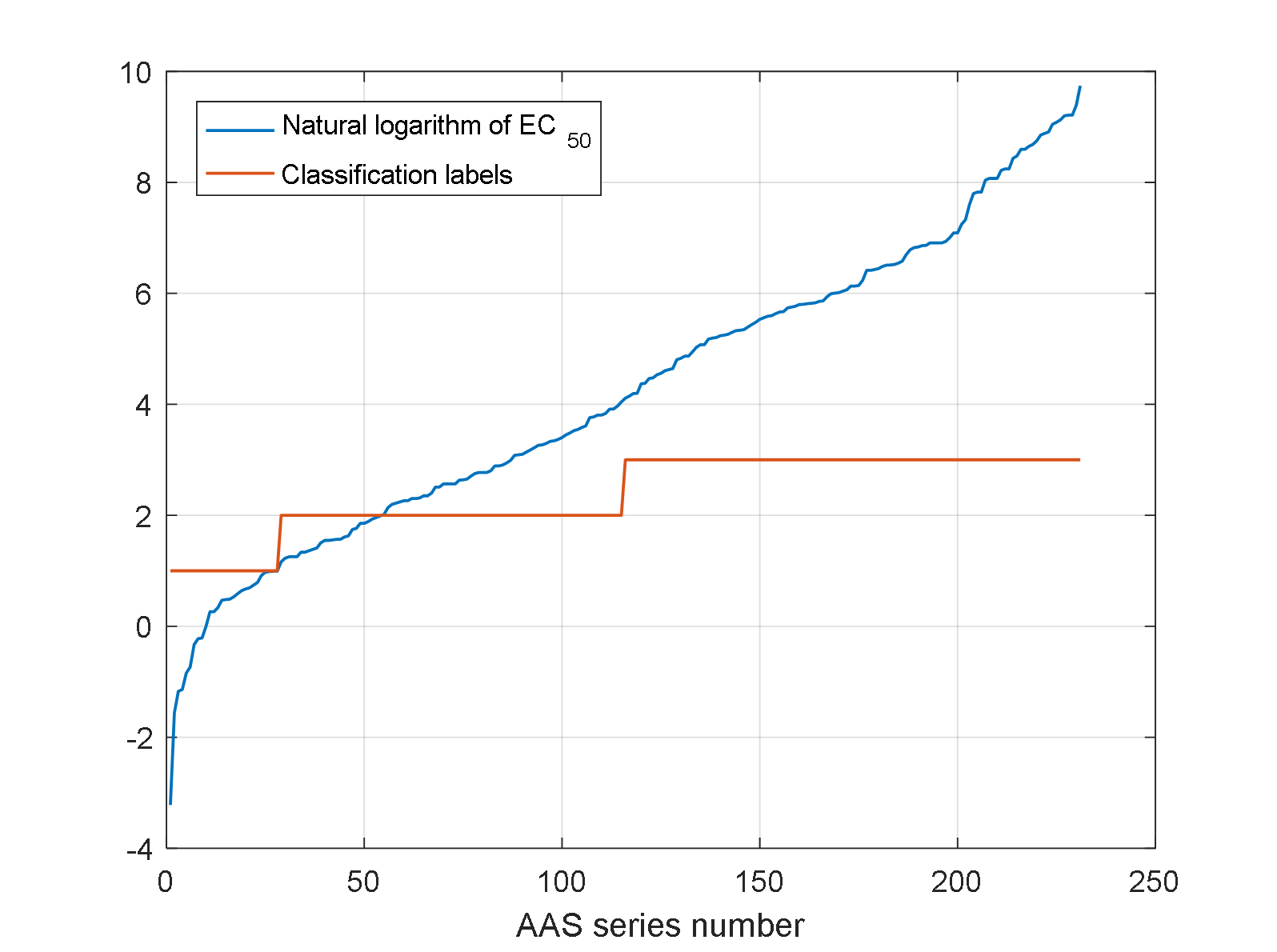

Supplement: Supplementary file 2 [file DataSheet1.zip › figures/fig14b.tif]

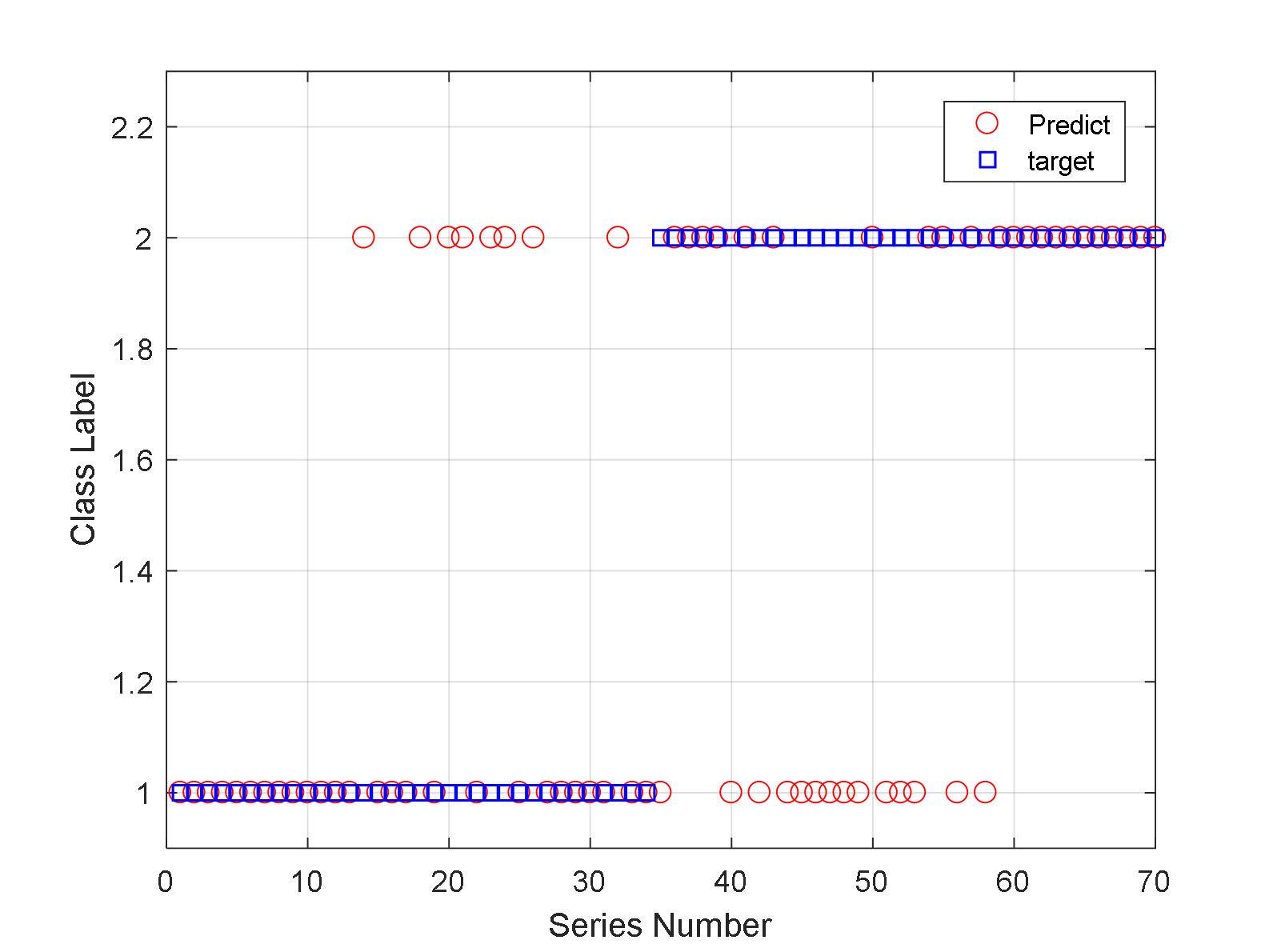

Supplement: Supplementary file 2 [file DataSheet1.zip › figures/fig15a.tif]

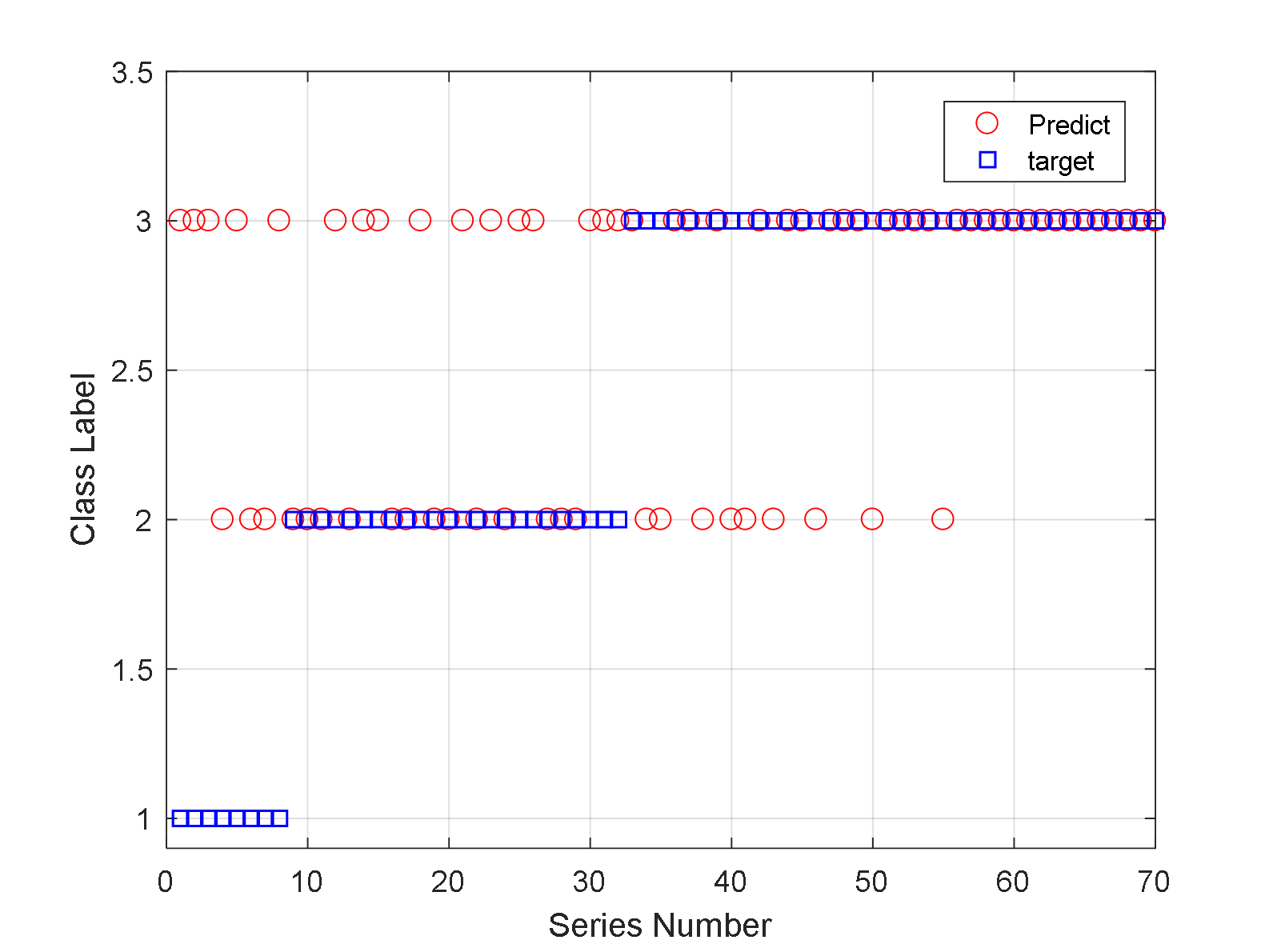

Supplement: Supplementary file 2 [file DataSheet1.zip › figures/fig15b.tif]

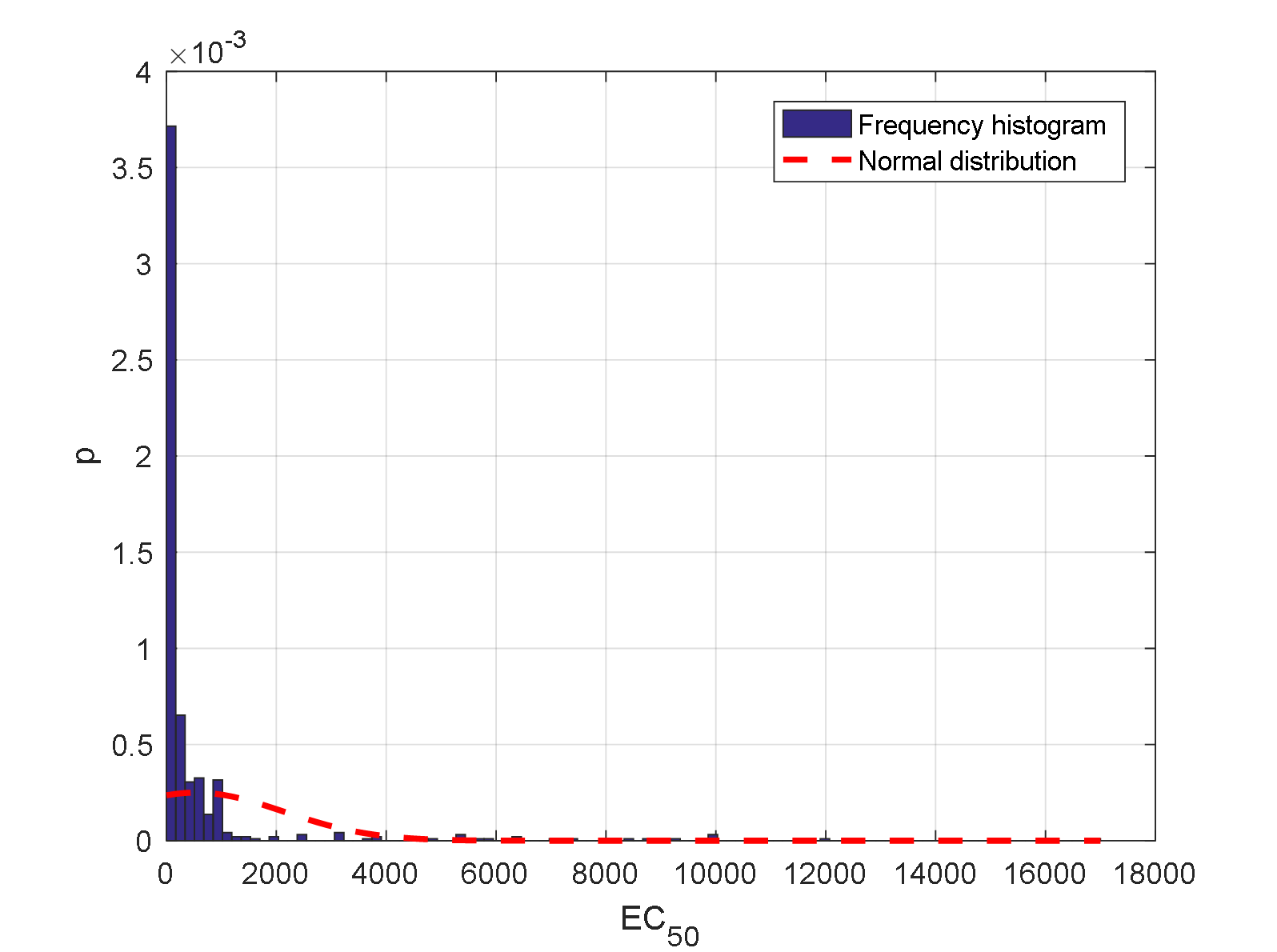

Supplement: Supplementary file 2 [file DataSheet1.zip › figures/fig2a.tif]

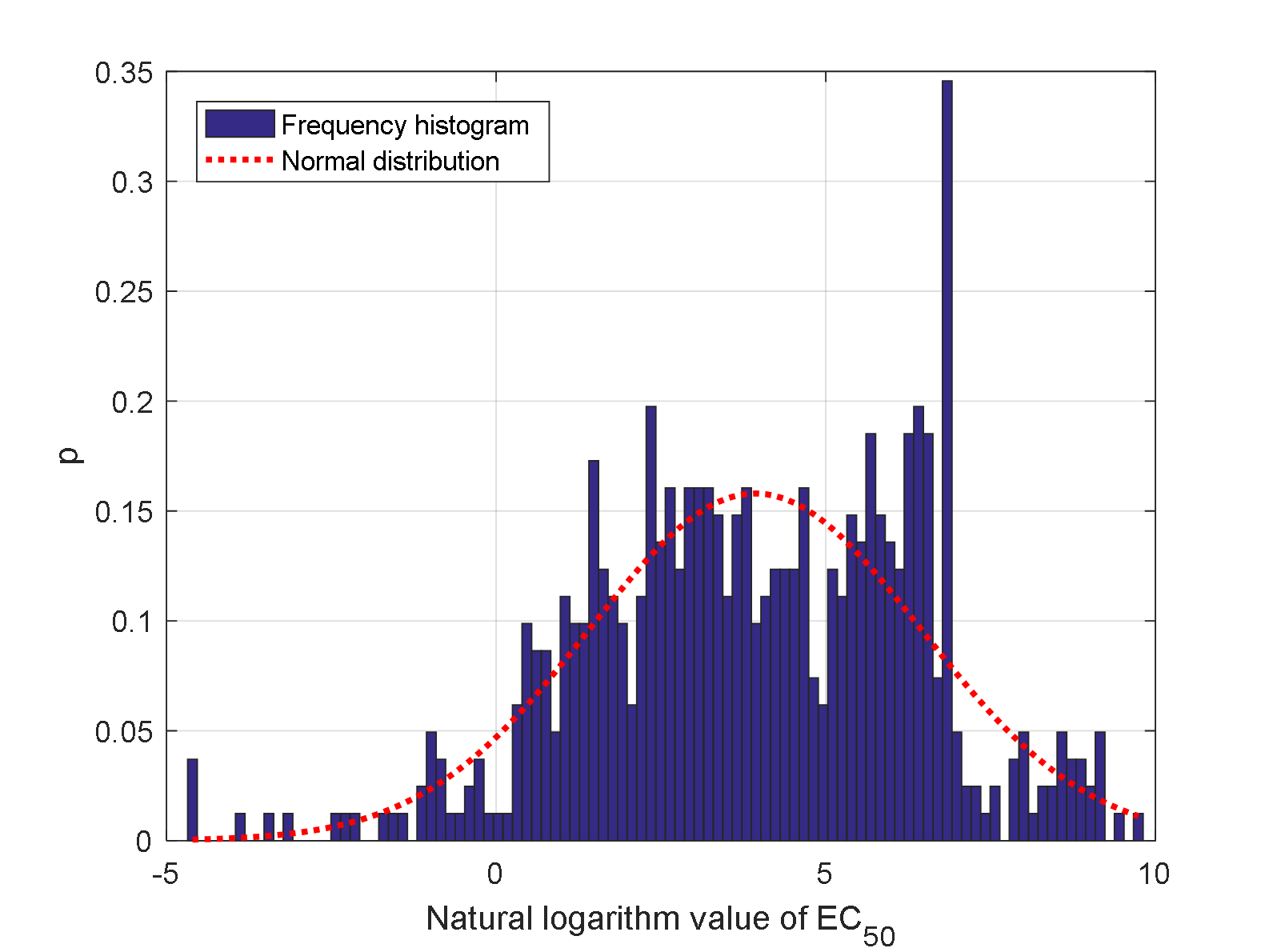

Supplement: Supplementary file 2 [file DataSheet1.zip › figures/fig2b.tif]

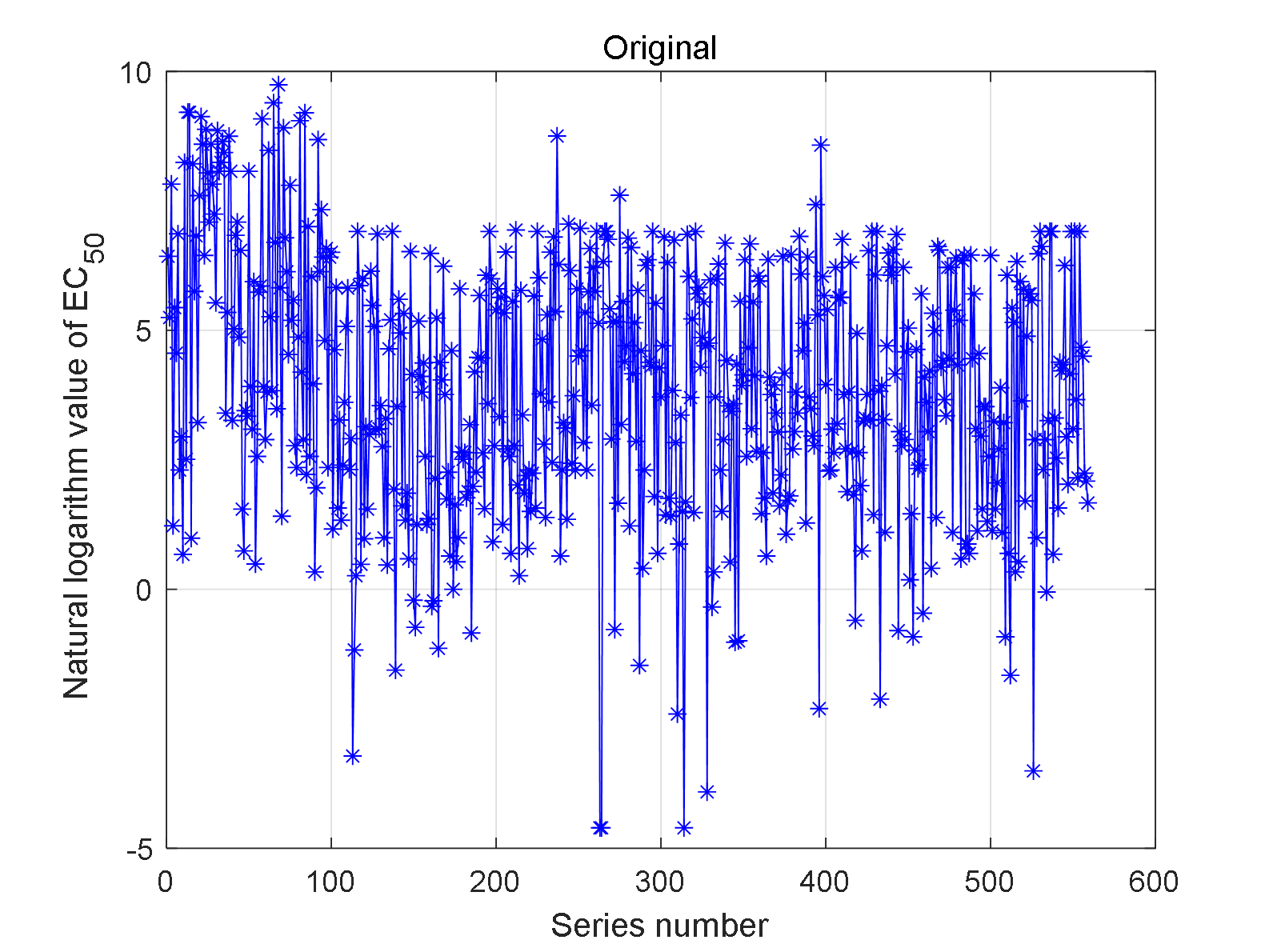

Supplement: Supplementary file 2 [file DataSheet1.zip › figures/fig3a.tif]

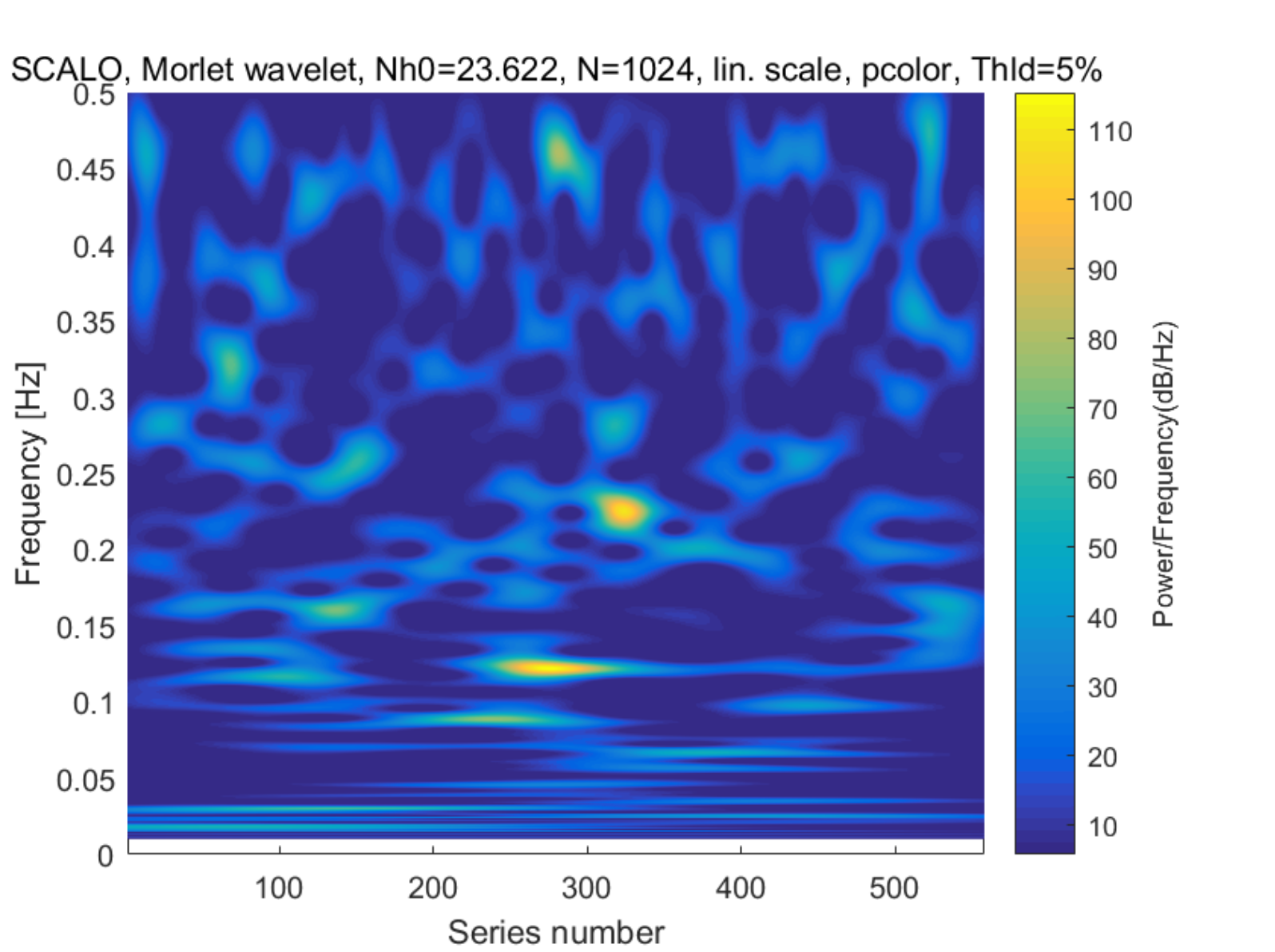

Supplement: Supplementary file 2 [file DataSheet1.zip › figures/fig3b.tif]

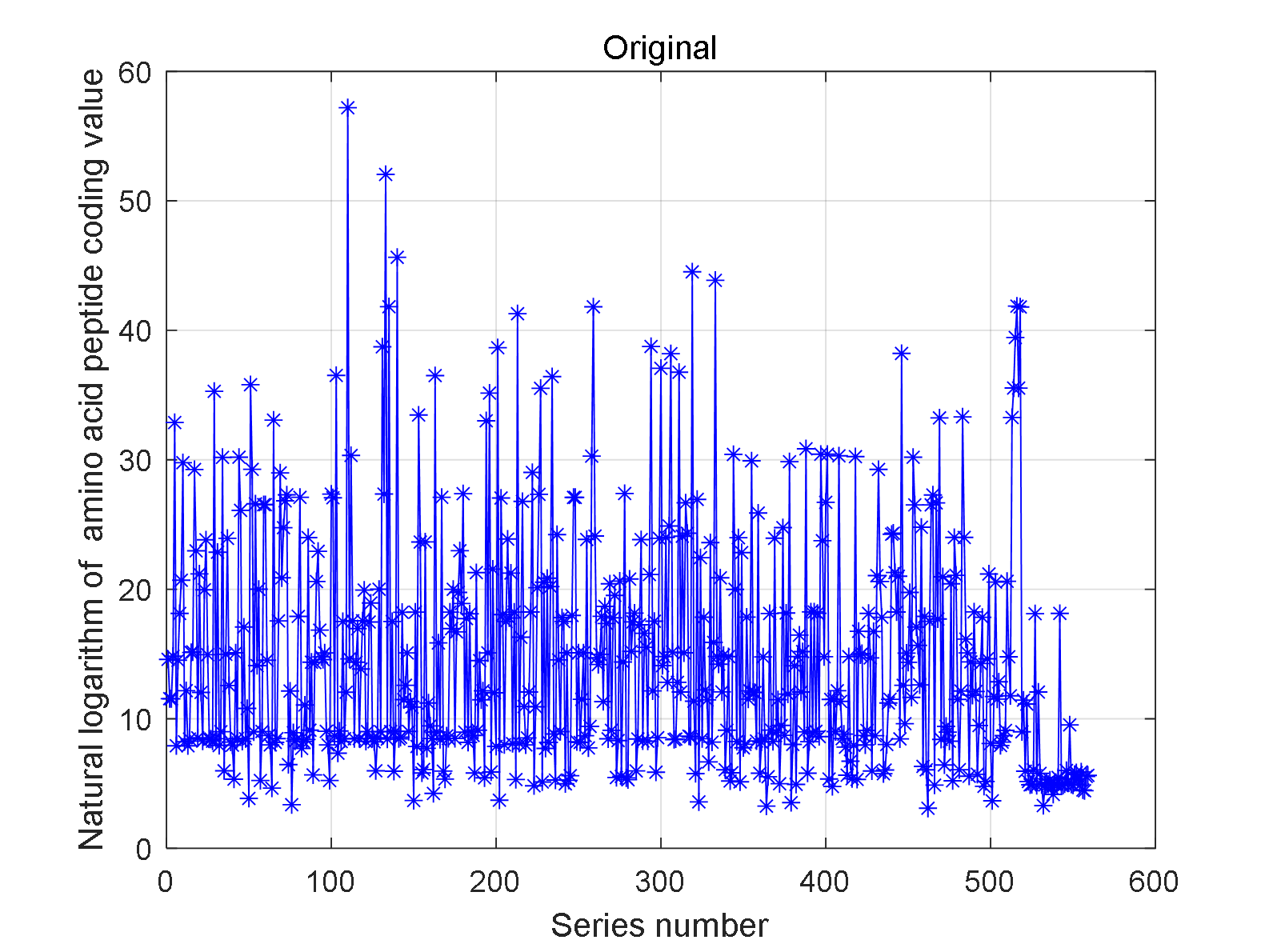

Supplement: Supplementary file 2 [file DataSheet1.zip › figures/fig4a.tif]

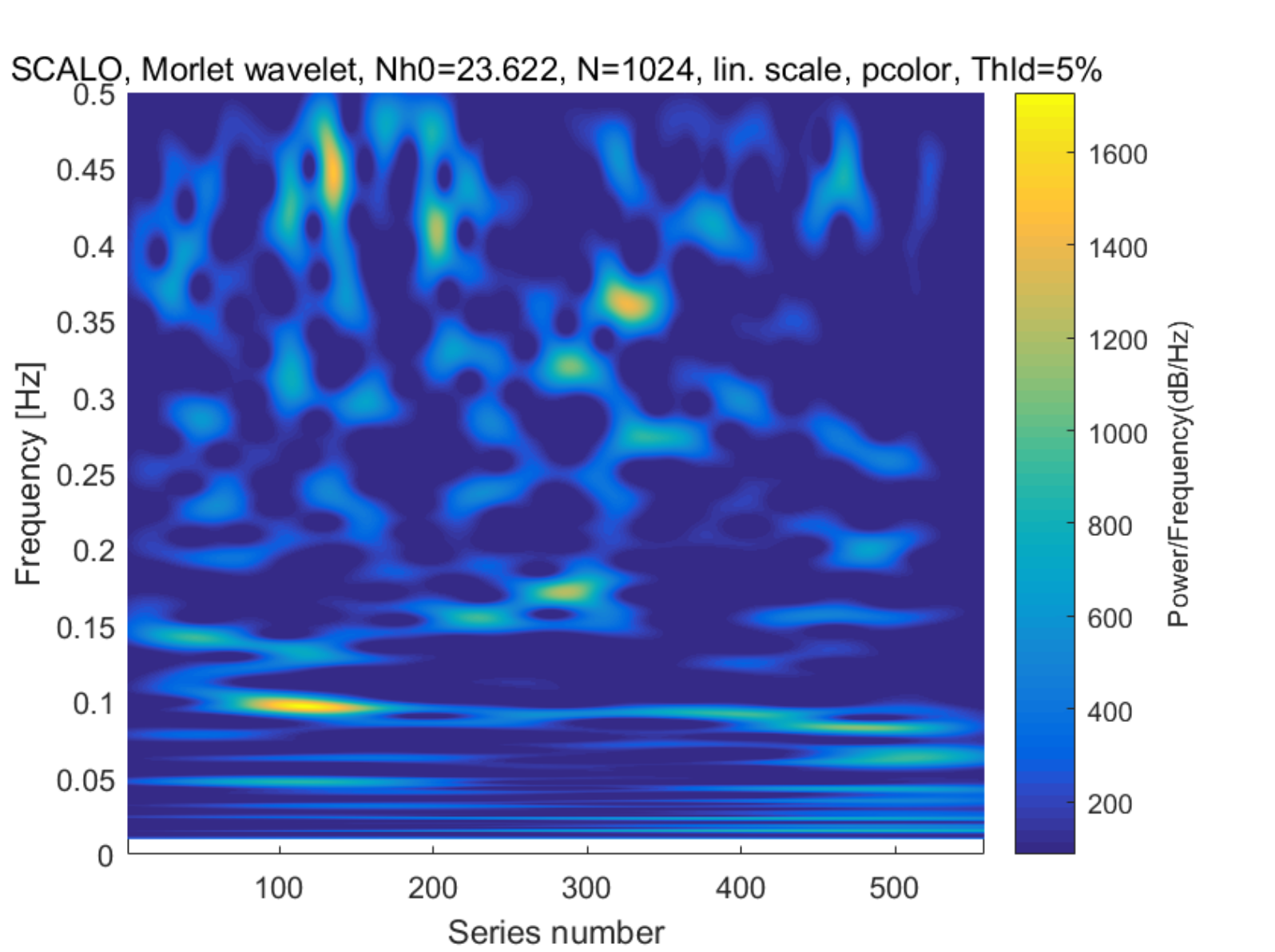

Supplement: Supplementary file 2 [file DataSheet1.zip › figures/fig4b.tif]

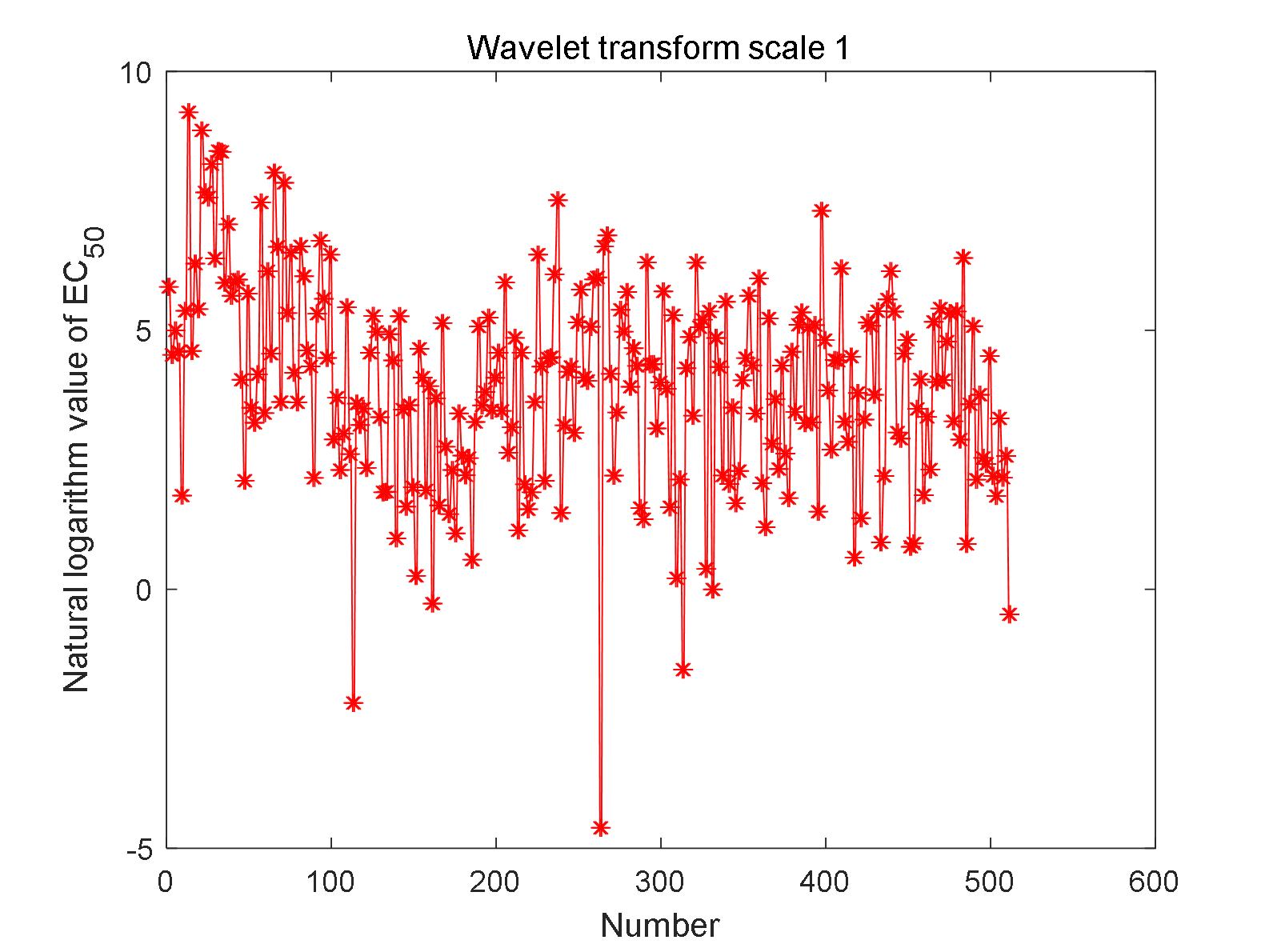

Supplement: Supplementary file 2 [file DataSheet1.zip › figures/fig5a.tif]

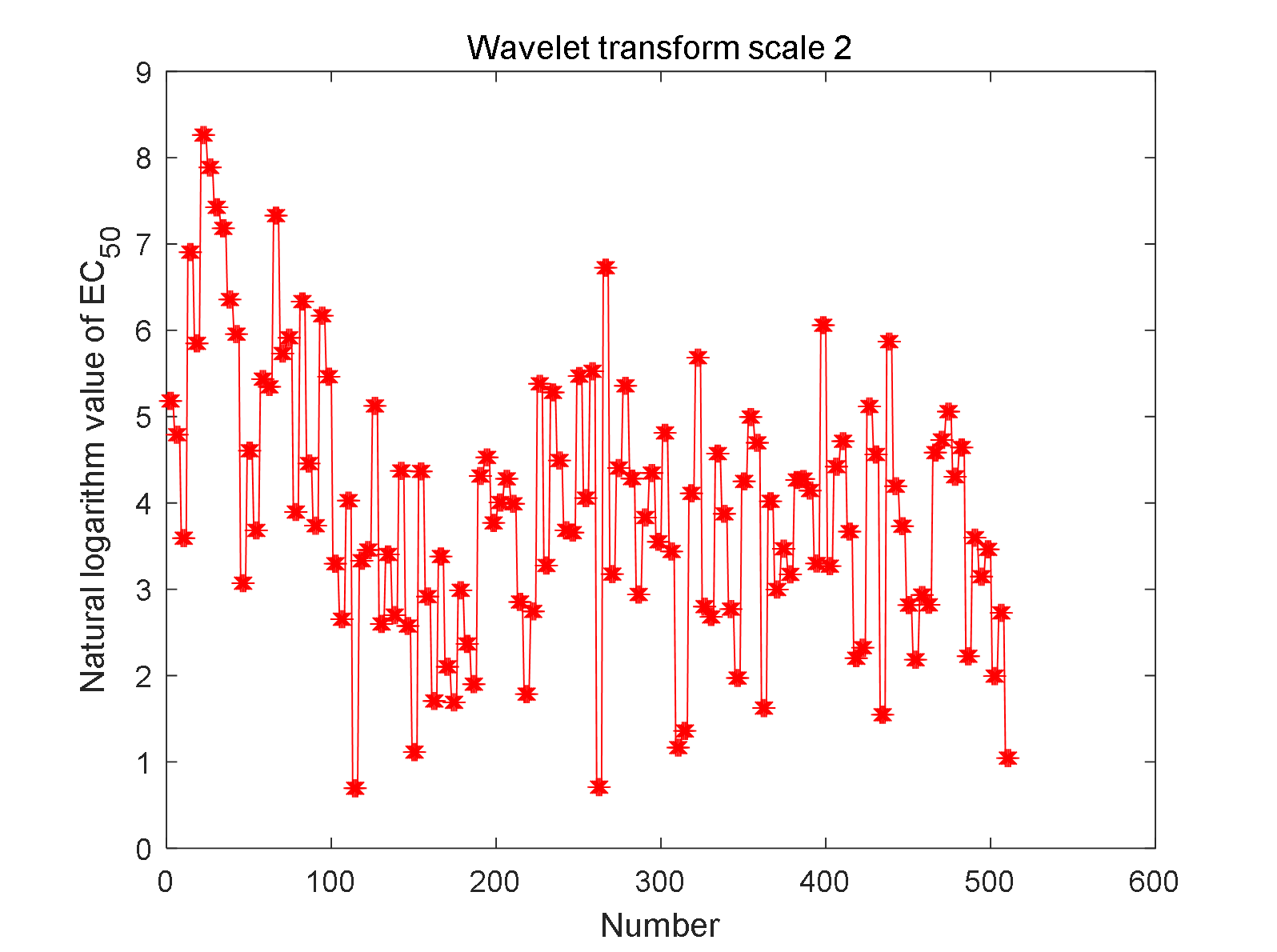

Supplement: Supplementary file 2 [file DataSheet1.zip › figures/fig5b.tif]

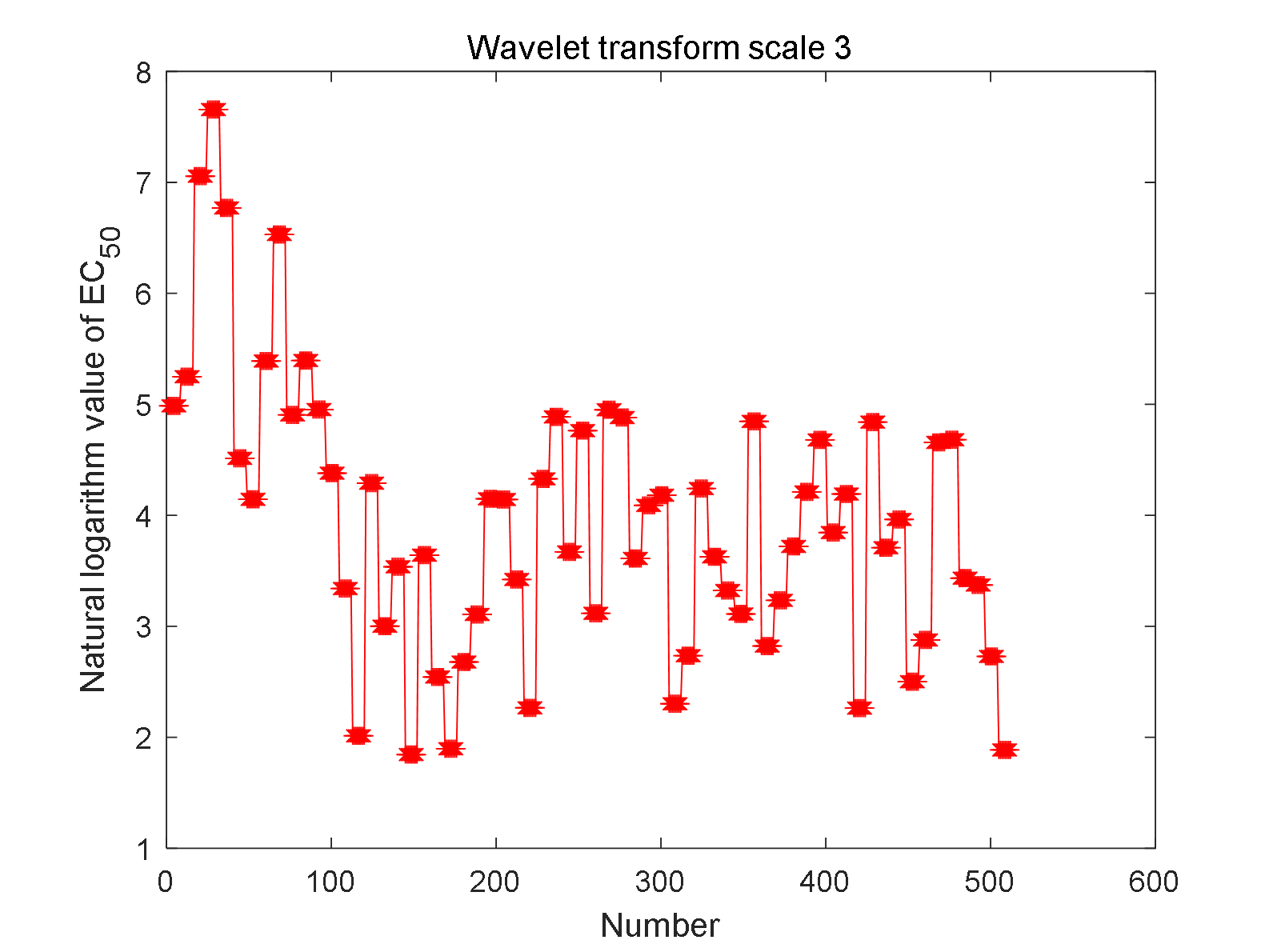

Supplement: Supplementary file 2 [file DataSheet1.zip › figures/fig5c.tif]

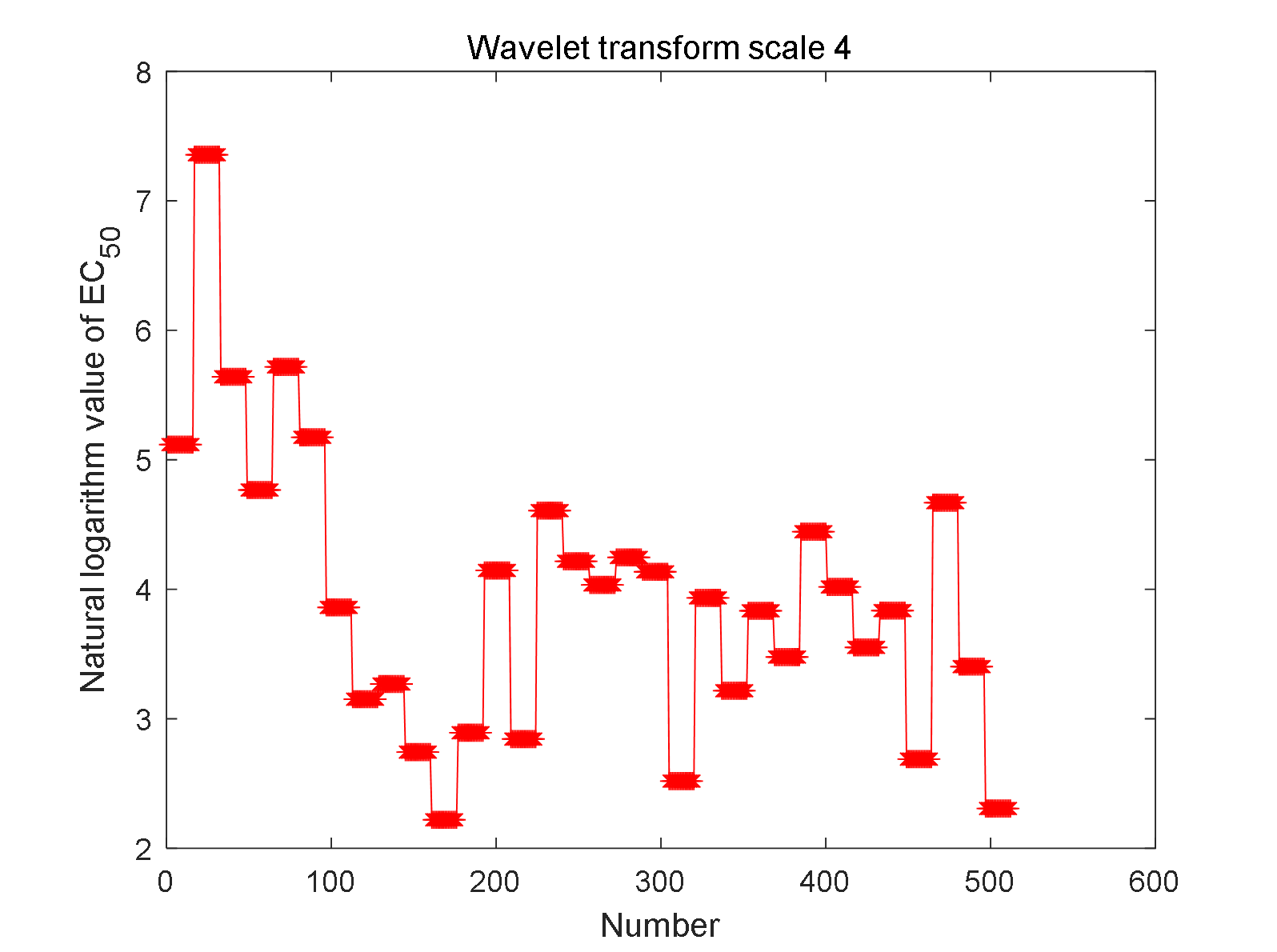

Supplement: Supplementary file 2 [file DataSheet1.zip › figures/fig5d.tif]

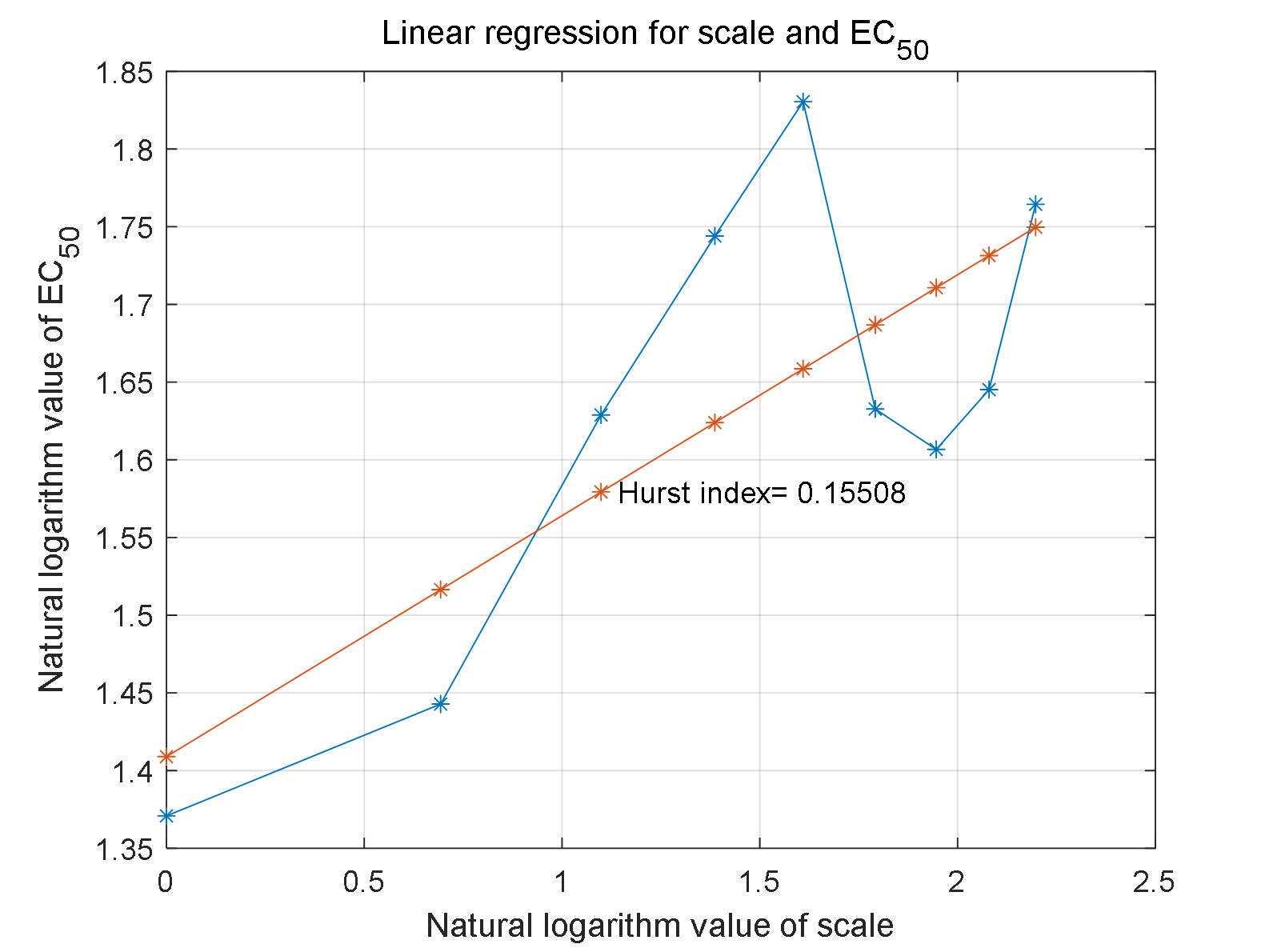

Supplement: Supplementary file 2 [file DataSheet1.zip › figures/fig6a.tif]

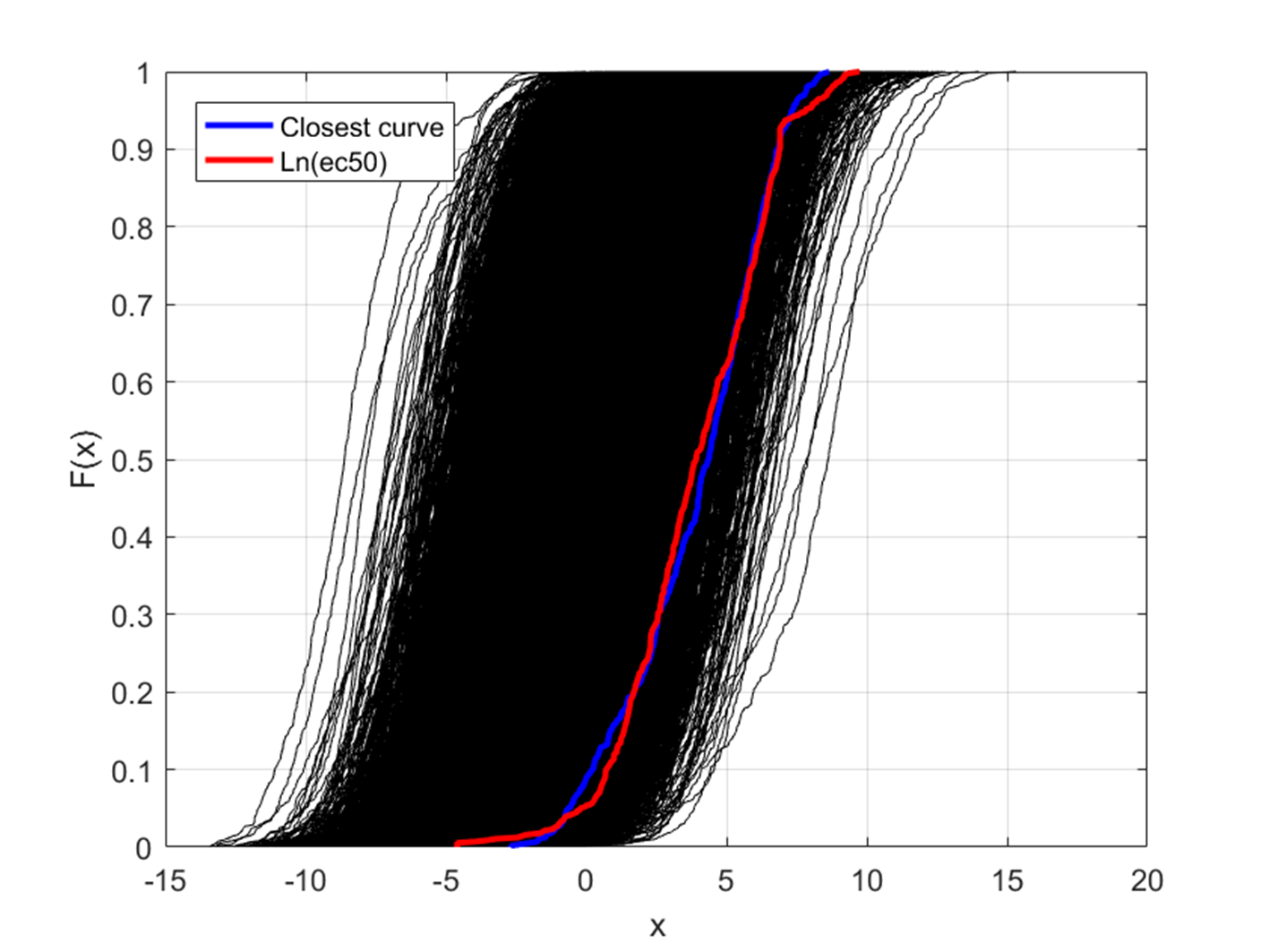

Supplement: Supplementary file 2 [file DataSheet1.zip › figures/fig6b.tif]

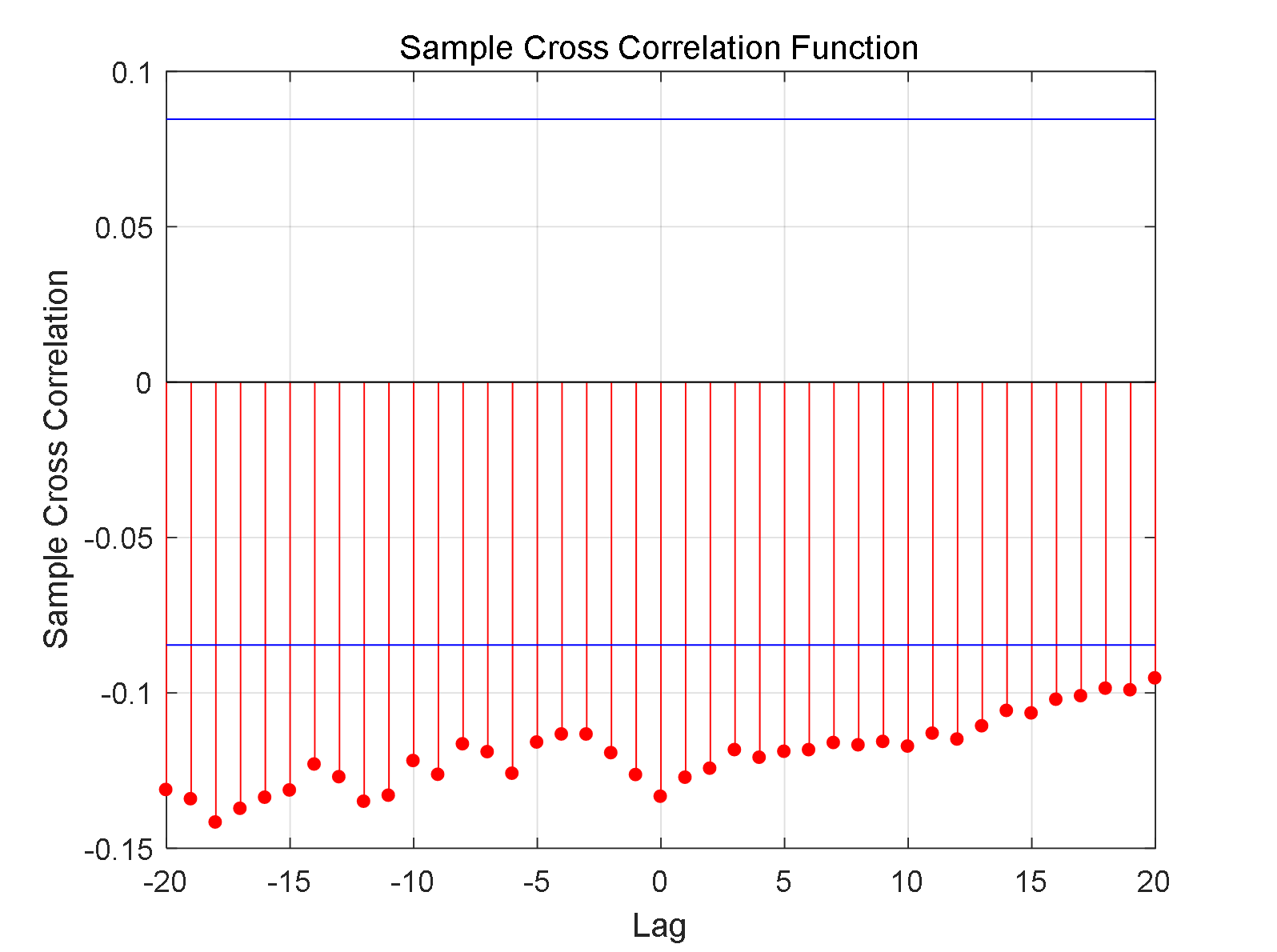

Supplement: Supplementary file 2 [file DataSheet1.zip › figures/fig7a.tif]

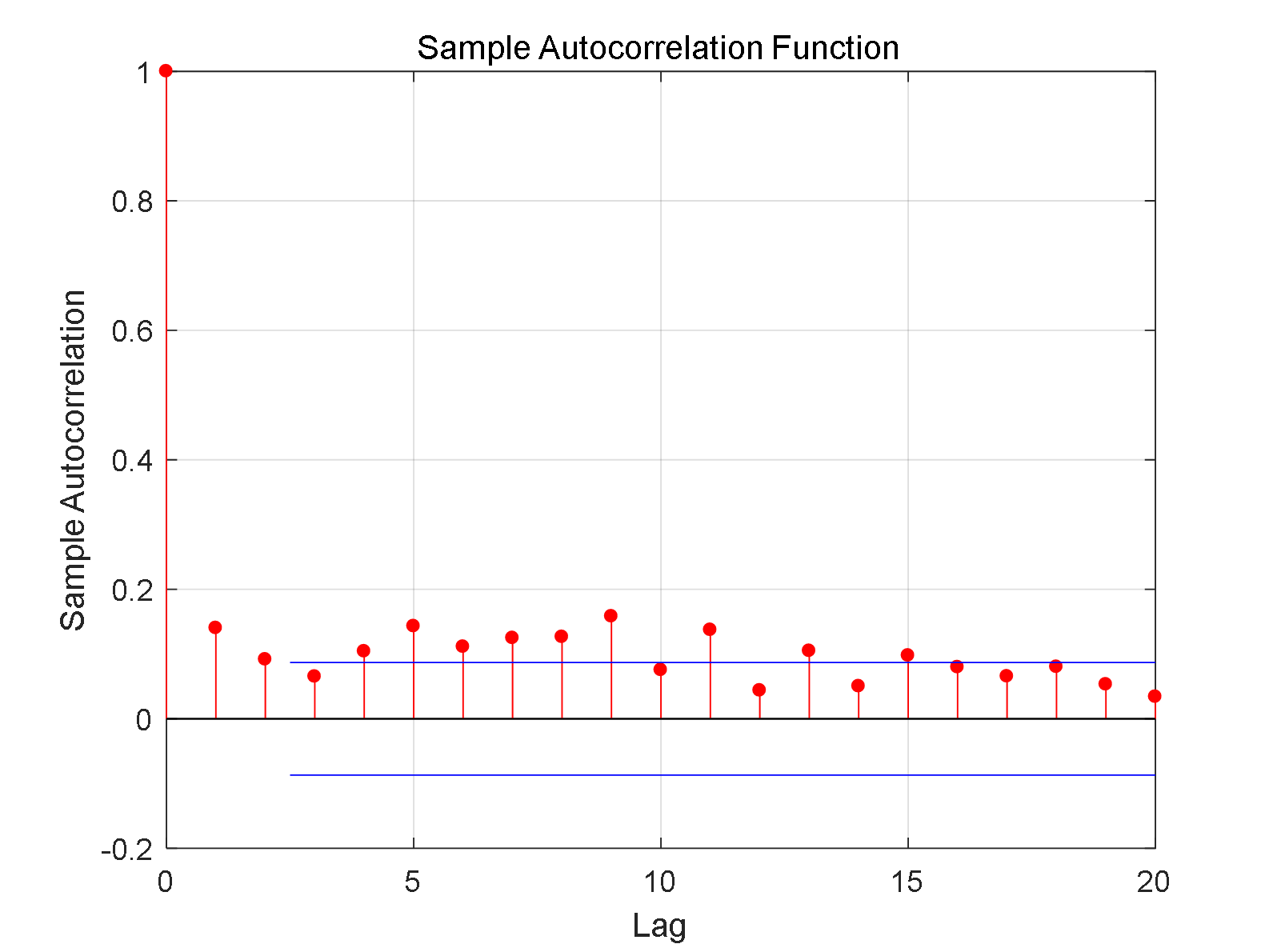

Supplement: Supplementary file 2 [file DataSheet1.zip › figures/fig7b.tif]

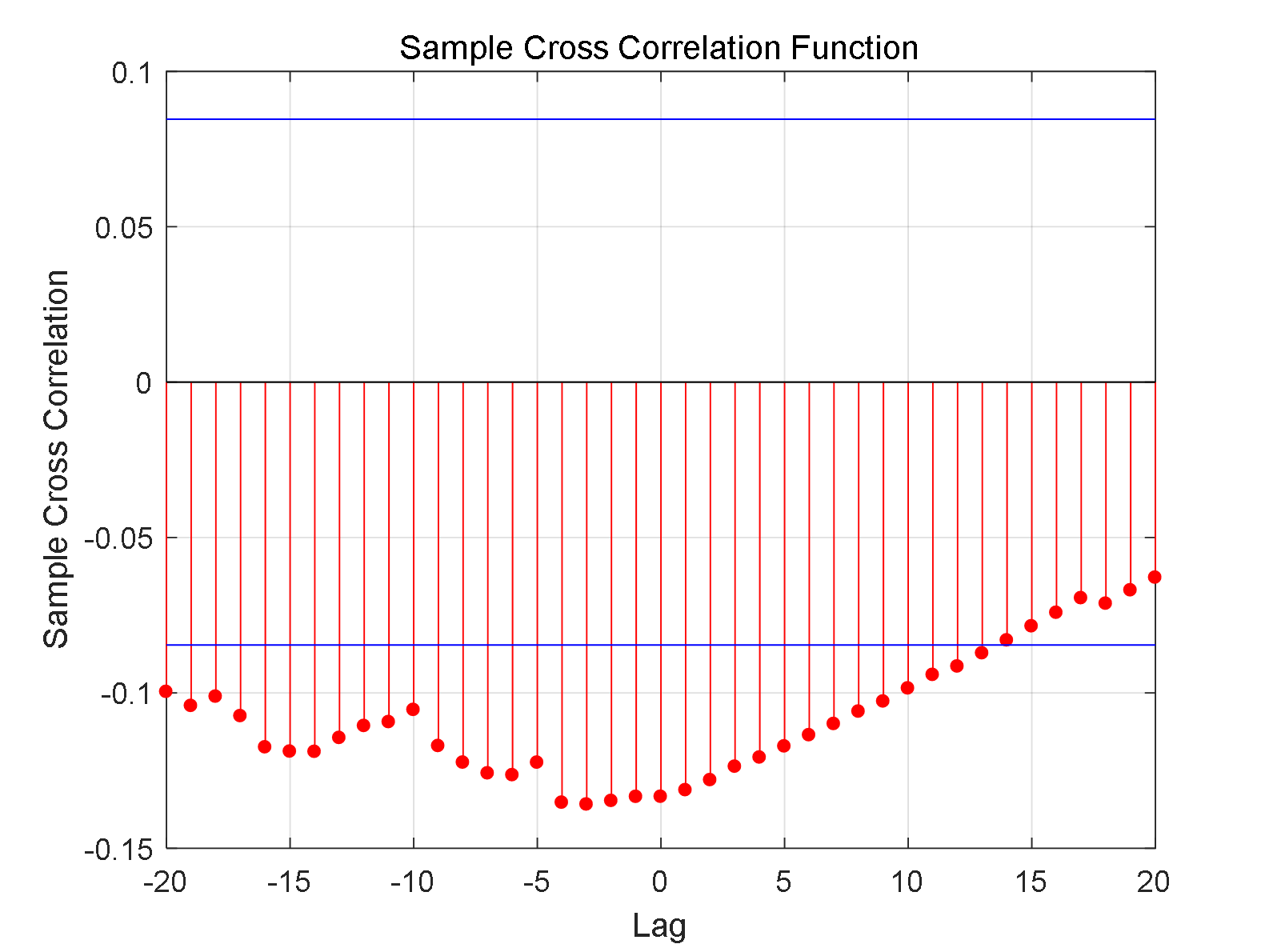

Supplement: Supplementary file 2 [file DataSheet1.zip › figures/fig8a.tif]

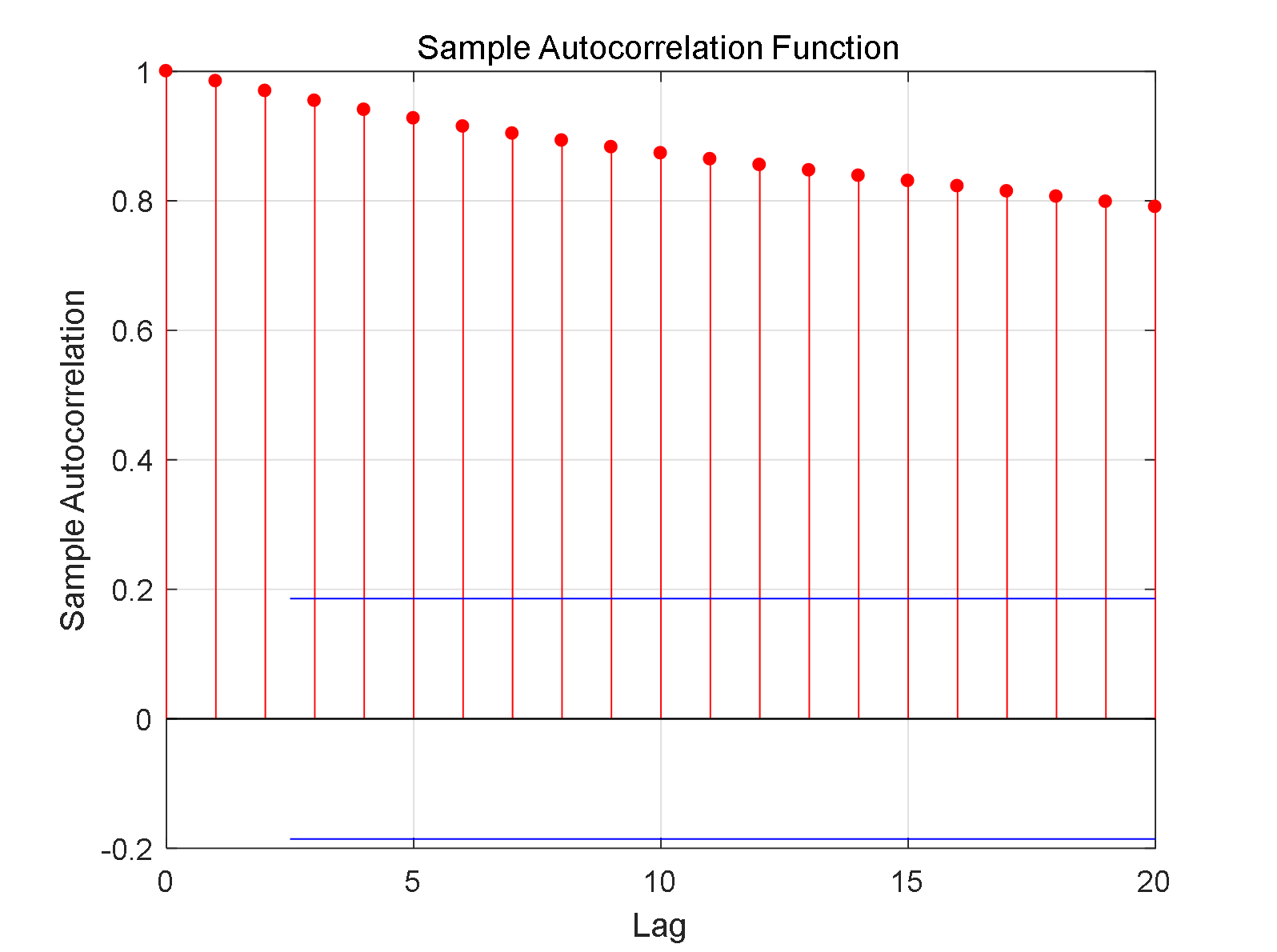

Supplement: Supplementary file 2 [file DataSheet1.zip › figures/fig8b.tif]

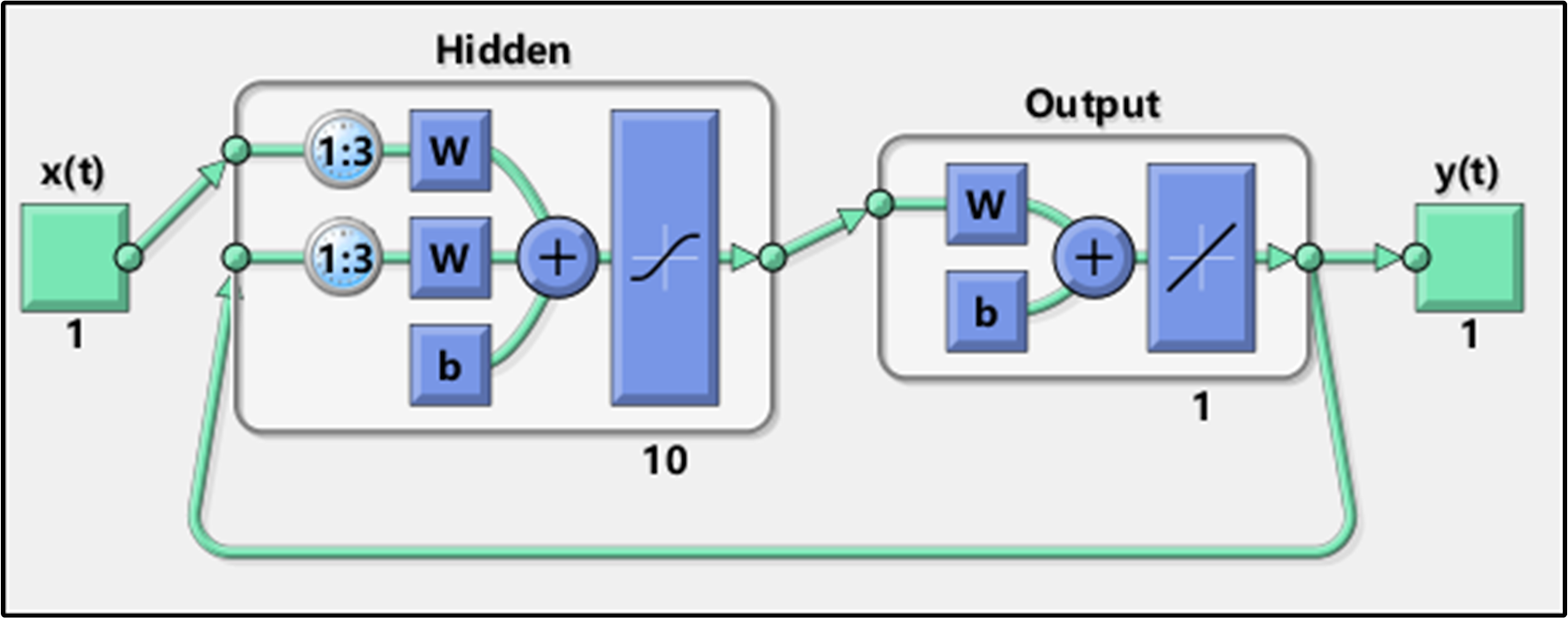

Supplement: Supplementary file 2 [file DataSheet1.zip › figures/fig9a.tif]

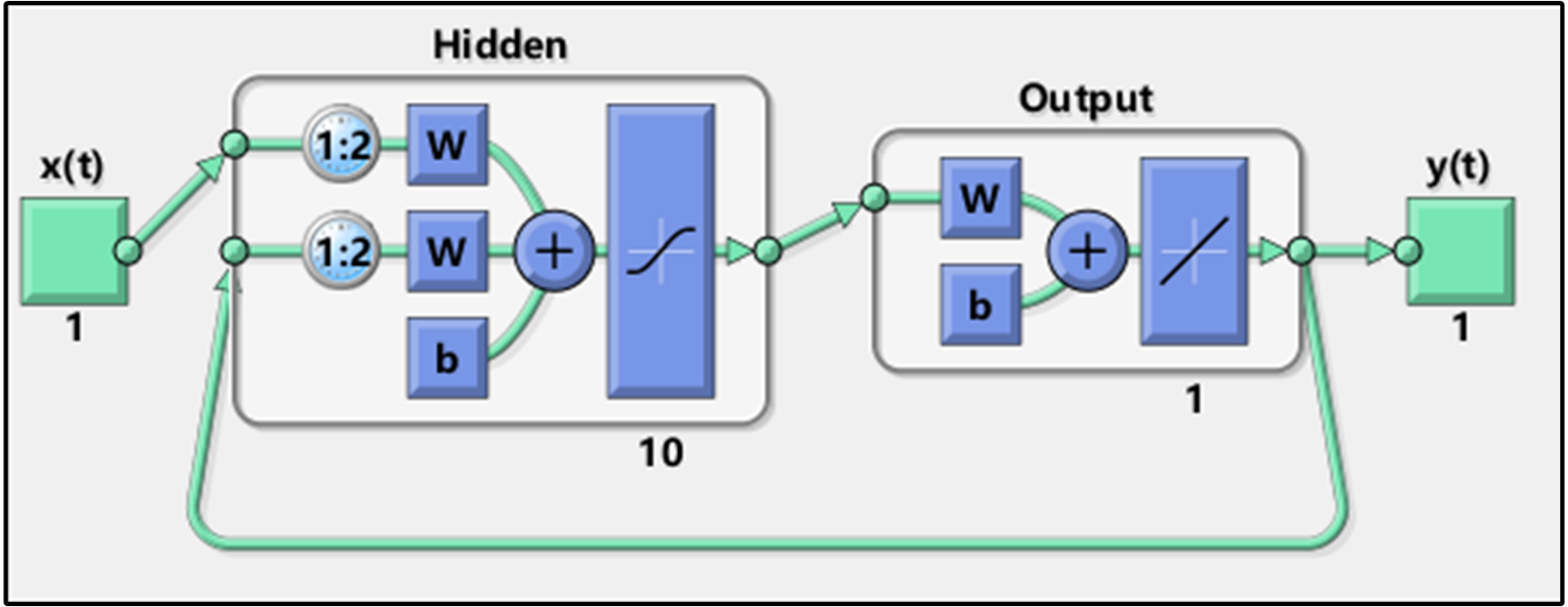

Supplement: Supplementary file 2 [file DataSheet1.zip › figures/fig9b.tif]
